# Association between sleep duration and metabolic syndrome: linear and nonlinear Mendelian randomization analyses

**DOI:** 10.1186/s12967-023-03920-2

**Published:** 2023-02-07

**Authors:** Yannis Yan Liang, Jie Chen, Miaoguan Peng, Jiajin Zhou, Xinru Chen, Xiao Tan, Ningjian Wang, Huan Ma, Lan Guo, Jihui Zhang, Yun-Kwok Wing, Qingshan Geng, Sizhi Ai

**Affiliations:** 1 Guangdong Cardiovascular Institute, Guangdong Provincial People’s Hospital, Guangdong Academy of Medical Sciences, 102 Zhongshan Road, Guangzhou, Guangdong China; 2 Li Chiu Kong Family Sleep Assessment Unit, Department of Psychiatry, Faculty of Medicine, The Chinese University of Hong Kong, Shatin, Hong Kong, SAR China; 3 Department of Endocrinology, The Third Affiliated Hospital of Guangzhou Medical University, Guangzhou, Guangdong China; 4 The Affiliated Hospital of Kunming University of Science and Technology, The First People’s Hospital of Yunnan Province, Kunming, Yunnan China; 5 The First School of Clinical Medicine, Southern Medical University, Guangzhou, China; 6 Department of Big Data in Health Science, Zhejiang University School of Public Health, Hangzhou, China; 7 Department of Psychiatry, Sir Run Run Shaw Hospital, Zhejiang University School of Medicine, Hangzhou, China; 8 Department of Medical Sciences, Uppsala University, Uppsala, Sweden; 9 Institute and Department of Endocrinology and Metabolism, Shanghai Ninth People’s Hospital, Shanghai JiaoTong University School of Medicine, Shanghai, China; 10 Guangdong Provincial Key Laboratory of Coronary Heart Disease Prevention, Guangdong Cardiovascular Institute, Guangdong Provincial People’s Hospital, Guangdong Academy of Medical Sciences, Guangzhou, Guangdong China; 11 Center for Sleep and Circadian Medicine, The Affiliated Brain Hospital of Guangzhou Medical University, 36 Mingxin Road, Guangzhou, Guangdong China; 12 Department of Geriatrics, Shenzhen People’s Hospital, The Second Clinical Medical College, Jinan University, Shenzhen, Guangdong China; 13 Department of Geriatrics, The First Affiliated Hospital, Southern University of Science and Technology, Shenzhen, Guangdong China; 14 Department of Cardiology, Life Science Center, Heart Center, The First Affiliated Hospital of Xinxiang Medical University, Weihui, Henan China

**Keywords:** Mendelian randomization, Sleep duration, Metabolic syndrome, Hyperglycemia, Obesity

## Abstract

**Background:**

Observational studies have found that both short and long sleep duration are associated with increased risk of metabolic syndrome (MetS). This study aimed to examine the associations of genetically determined sleep durations with MetS and its five components (i.e., central obesity, high blood pressure, dyslipidemia, hypertriglyceridemia, and hyperglycemia) among a group of elderly population.

**Methods:**

In 335,727 participants of White British from the UK Biobank, linear Mendelian randomization (MR) methods were first employed to examine the causal association of genetically predicted continuous sleep duration with MetS and its each component. Nonlinear MR analyses were performed to determine the nonlinearity of these associations. The causal associations of short and long sleep duration with MetS and its components were further assessed by using genetic variants that associated with short (≤ 6 h) and long sleep (≥ 9 h) durations.

**Results:**

Linear MR analyses demonstrated that genetically predicted 1-h longer sleep duration was associated with a 13% lower risk of MetS, a 30% lower risk of central obesity, and a 26% lower risk of hyperglycemia. Non-linear MR analyses provided evidence for non-linear associations of genetically predicted sleep duration with MetS and its five components (all P values < 0.008). Genetically predicted short sleep duration was moderately associated with MetS and its four components, including central obesity, dyslipidemia, hypertriglyceridemia, and hyperglycemia (all P values < 0.002), whereas genetically long sleep duration was not associated with MetS and any of its components.

**Conclusions:**

Genetically predicted short sleep duration, but not genetically predicted long sleep duration, is a potentially causal risk factor for MetS.

**Graphical Abstract:**

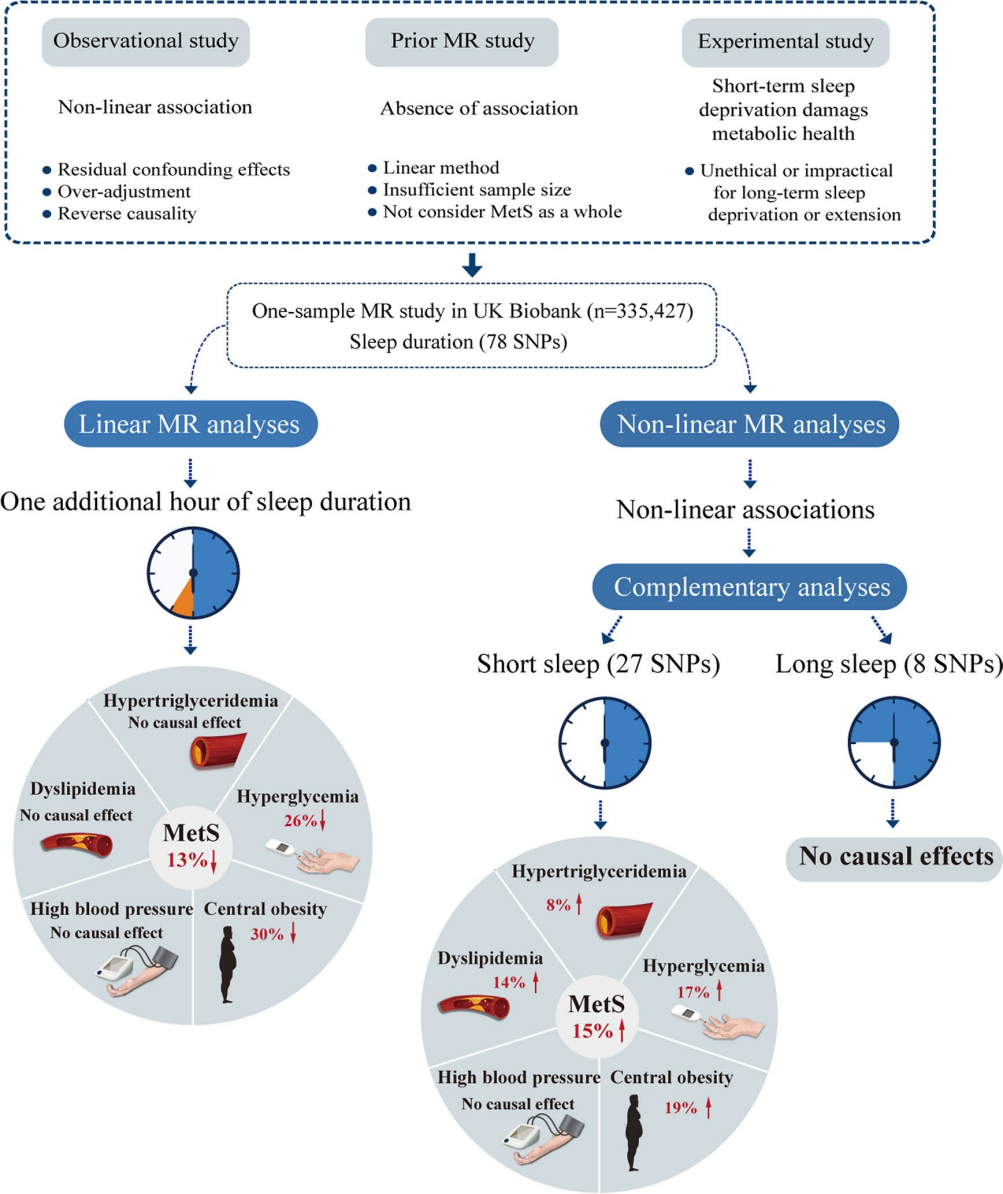

**Supplementary Information:**

The online version contains supplementary material available at 10.1186/s12967-023-03920-2.

## Background

Central obesity, high blood pressure, dyslipidemia, hypertriglyceridemia, and hyperglycemia, as well as metabolic syndrome (MetS) are prevalent in middle-aged and elderly population. Specifically, MetS is defined as having three out of the above five components [1]. The global prevalence of Mets is 20–25% in the adult population but varied across different countries, for example, the estimated prevalence of MetS is 33.4% in the United States, while it is 14.4% in China [2–4]. MetS dramatically increase the risk of developing cardiovascular diseases, which constitute by far the leading cause of morbidity and mortality worldwide [1]. Thus, it is warranted to identify its causal risk factors for prevention and treatment. In the past two decades, emerging observational studies have found that both short and long sleep duration are associated with MetS and its components (e.g., central obesity and hyperglycemia) [5–9], which suggests that usual habitual sleep duration may serve as a possible risk factor and preventive target of MetS.

However, observational studies are vulnerable to residual confounding effect, over-adjustment of potential mediators, and reverse causality [10]. Therefore, observational study is less powerful to examine causality between exposure and outcome. These limitations can, of course, be tackled by implementing a randomized controlled trial (RCT) or experimental study. As summarized before, considerable experimental studies have consistently found that short-term sleep deprivation causes a series of metabolic changes, including impaired glucose and lipid metabolism, dysregulated neuroendocrine systems, and increased energy intake [11–13]. However, it seems impractical to experimentally extend one’s sleep duration for the long term among the individuals without sleep loss. Hence, the evidence supporting the detrimental effects of habitual long sleep duration on metabolic health was lack. Finally, it is unethical to conduct experiment of long-term sleep deprivation or sleep extension to confirm the causal effect of unusual sleep duration on MetS.

Mendelian randomization (MR), which leverages the genetic variants (randomly allocated from parents to offspring) as proxies for life-long exposure risks, is able to reduce bias from confounding factors and reverse causation [10]. Some pioneer two-sample MR studies have addressed potential causal links between sleep duration and some single metabolic component, such as adiposity in children [14] and type 2 diabetes [15]; in contrast, most of the existing MR studies failed to draw causal effects of genetically predicted sleep duration on metabolic outcomes [9, 16–21]. Notably, these MR studies have not considered the potential non-linear associations between sleep duration and MetS as indicated by previous epidemiological studies [5, 22]. Non-linear MR is a recently developed state-of-the-art approach, which generated localized average causal effect (LACE) estimates by conditioning on quantiles of instrumental variable (IV)-free exposure [23, 24]. This method can evaluate the potential nonlinearity between risk factor and disease outcomes. Our previous MR study has used this novel method to address the nonlinear association between sleep duration and a series of cardiovascular disorders [25]. However, to our knowledge, there is no study that has attempted to clarify the potential nonlinear MR associations of sleep duration with MetS and its components.

Thus, the present study aimed to examine the potential casual effects of genetically predicted sleep duration (short and long) on the risk of MetS and its components by using linear and non-linear MR methods. First, we investigated whether continuous sleep duration causally influenced the risk of MetS using linear MR analyses. Second, we delineated the shape of causal relationship between genetically predicted sleep duration and MetS using non-linear MR analyses. Last, we determined how genetically predicted short and long sleep durations are causally associated with MetS and each of its components, respectively, with using genetic variants associated with short and long sleep durations.

## Methods

### Data sources and study participants

This study used data from the UK Biobank, which is a population-based cohort study that enrolled more than 500,000 participants aged 40 to 69 years in 22 assessment centers across the UK between 2006 and 2010. All participants have given informed consent to participate in this cohort study. The present study initially included 400,778 participants with valid measure for both sleep duration and MetS in the UK Biobank at the time of the current study. The quality control process of genetic data was listed as follow: (a) without genetic data; (b) non-European ancestry based on questionnaire and principal components analysis; (c) > 10 third-degree relatives identified or excluded from kinship inference process; (d) missing rates of high-quality markers; (e) outliers for heterozygosity or missing rate; (f) sex mismatch. Finally, a total of 335,727 participants with a mean age of 56.9 year were included in the analyses. The flow chart of participant selection is available in Additional file 1: Fig. S1*.* UK Biobank has received ethical approval from the UK National Health Service’s National Research Ethics Service (ref 11/NW/0382).

### Ascertainment of exposure, outcomes, and covariates

Self-reported habitual sleep duration was the major exposure of the current study. It was obtained from touchscreen questionnaires at baseline assessment. Sleep duration was assessed with a standardized question: “About how many hours sleep do you get in every 24 h? (Please include naps)” (field ID: 1160). Participants who answered “Do not know” and “Prefer not to answer”, and those provided implausible sleep durations (< 4 h or > 11 h per day) were excluded to minimize implausible sleep duration and potential confounding by poor health. Continuous sleep duration was categorized into three groups: short (≤ 6 h per day), normal (7 to 8 h per day), and long (≥ 9 h per day) sleep durations corresponding to the classification criteria from a prior GWAS study about sleep duration in UK Biobank [26].

MetS were defined as the presence of having three or more of the following components: central obesity, high blood pressure, dyslipidemia, hypertriglyceridemia, and hyperglycemia [1]. These measures were assessed at the initial visit. The definitions of each component were listed as follow: (a) waist circumference (Field ID 48) was measured using Seca 200 cm tape passing around the smallest part of the trunk. The cutoff points for defining central obesity were ≥ 88 cm for women and ≥ 102 cm for men; (b) Systolic and diastolic pressures (Field ID 4079, 94, 4080, 93) were measured twice using IntelliSense blood pressure monitor model HEM-907XL (Omron) after a rest of at least 5 min. High blood pressure was defined as a systolic blood pressure of 130 mmHg or greater (for both times), or a diastolic blood pressure of 85 mmHg or greater (for both times), or as taking antihypertensive drugs; (c) Low high-density lipoprotein (HDL) cholesterol (Field ID 30760) was defined as either low HDL (< 1.0 mmol/L (male), < 1.3 mmol/L (female)), or taking drugs treating low HDL cholesterol; (d) Hypertriglyceridemia was defined as a level of triglycerides ≥ 1.7 mmol/L (Field ID 30870) or taking triglyceride-lowering drugs; (e) Hyperglycemia was diagnosed if hemoglobin A1c (HbA1c) (Field ID 30750) was ≥ 42 mmol/mol (6.0%), or taking medication for diabetes. The information of the above-mentioned drugs is listed in Additional file 1: Table S1.

We considered several factors to be potential confounders of the association between sleep duration and MetS risk: age (field ID 21022), sex (field ID 31), education (field ID 6138), employment (field ID 6142), smoking status (field ID 20116), drinking status (field ID 1558), socioeconomic status (Townsend deprivation index, TDI, field ID 189), and physical activity (field ID 22032). However, physical activity was not used in our final analysis due to the extensive missing numbers. To consider the potential effects of other sleep traits on the MetS risk, we also included insomnia (field ID 1200), daytime napping (field ID 1190), chronotype (field ID 1180), snoring (field ID 1210), and daytime sleepiness (field ID 1220) as potential confounders. Those factors may associate with the genetic variants and confounded the exposure-outcome association. To test the potential violation of the MR assumption by those confounders, we investigated the associations between unweighted GRS and those factors in UK Biobank by linear regression model with a Bonferroni significance threshold of *P* < 0.004. We then performed sensitivity analyses in the one-sample linear MR analysis with further adjustment of those significant confounders.

### Generation of genetic risk score

We initially selected 78 single-nucleotide polymorphisms (SNPs) associated with continuous sleep duration at a genome-wide significance threshold (*P* < 5 × 10^–8^), corresponding to the recent GWAS study about self-reported sleep duration in the UK Biobank [26], as candidates to generate the genetic instruments (Additional file 1: Table S2). To minimize one-sample bias towards the confounded observational association, we summarized the number of sleep duration-increasing alleles to generate the unweighted genetic risk score (GRS) rather than weighted GRS [27, 28]. The unweighted GRS explained 0.62% of the variance in sleep duration by comparing residual variance in linear regression models of sleep duration on GRS (R^2^ = 0.62%, F-statistic = 2090). Next, in the complementary analyses, a total of 27 SNPs associated with short sleep duration (≤ 6 h per day) (Additional file 1: Table S3), and 8 SNPs associated with long sleep duration (≥ 9 h per day) (Additional file 1: Table S4) from the same GWAS study in the UK Biobank [26] were chosen to compute as the instrumental variables.

### Study design

We first investigated the association of unweighted GRS of self-reported sleep duration with MetS and its components using a simple method (Additional file 1: Text S1). Second, standard linear MR analyses were employed to assess the associations of genetically predicted sleep duration with MetS and its components. The estimate from the linear MR analyses represents the average change in each component due to per standard deviation increase in genetically predicted sleep duration. Further, non-linear MR analyses were performed to delineate the shape of the associations of genetically predicted sleep duration and metabolic outcomes. Subsequently, we explored how genetically predicted short and long sleep durations were causally associated with MetS and each of its components, respectively, with using genetic variants associated with short and long sleep durations. Last, we carried out a series of sensitivity analyses to further confirm our analyses (Additional file 1: Fig. S2)*.* Of note, we rescaled the causal estimates (OR, odds ratios for the outcomes) of continuous sleep duration by multiplying per-minute log odds ratios by 60, and the causal estimates for short and long sleep duration were rescaled to make it easier to be interpreted for per doubling of genetic liability for short or long sleep durations by multiplying the log ORs by 0.693 [29].

### Linear Mendelian randomization analyses

We first applied a two-stage method to estimate the causal effect of the exposure on the outcome under the framework of linear MR. In the first-stage analysis, we regressed the exposures on the unweighted GRS; in the second stage, we regressed the outcome on the fitted values of the exposure from the first stage. The regression models in both stages were adjusted with the confounders such as age, sex, assessment centers, top 10 principal components of ancestry, and genotyping arrays of the participants. An online tool (https://sb452.shinyapps.io/power/) was used to calculate the statistical power of the linear MR analyses (Additional file 1: Table S5).

To verify whether the linear MR analyses violated the assumption of directional horizontal pleiotropy (where the instrumental variables are associated with the study outcome via other pathways but not the exposure), we first assessed the validity of the genetic variants by testing the associations of potential confounders with the GRS, then, repeated linear MR analyses adjusting for these confounders. Further, we ran another three methods namely the inverse variance weighted (IVW), weighted median, and MR-Egger [30, 31] with ‘TwoSampleMR’ package in a single large dataset (Additional file 1: Text S2). In addition, RadialMR analyses using modified second-order weights were conducted to identify the potential outliers [32]. We adopted a level of 0.05 divided by the number of SNPs being used in the main analyses. Finally, we excluded those identified outliers and repeated our sensitivity analyses by using the TwoSample MR method.

### Non-linear Mendelian randomization analyses

A piecewise linear method in the non-linear MR framework was used to estimate the non-linearity of the causal effects of sleep duration on the risk of the disease outcomes [24]. Briefly, similar to the methods of previous studies [25, 26, 33], we first categorized our sample into three stratums by using residuals of the continuous sleep duration after regressing on the GRS. The adoption of three stratums was mainly due to the fact that sleep duration exposure was indeed a discrete variable (ranging from 4 to 11 h/day in the present study) rather than truly continuous one [33]. Second, we calculated the linear MR estimate in each stratum, which was confined to be continuous. We then used the gradient of each line segment as a localized average causal effect (LACE) in each stratum. Non-linearity was examined by the Quadratic test and the Cochran Q test [24].

### Complementary analyses

We summarized the short and long sleep duration risk alleles and weighted by its GWAS effect sizes, respectively. Next, we regressed them against the study outcomes (MetS and its five components) adjusting with age, sex, assessment center, top 10 principal components of ancestry, and genotyping array [34]. The effects estimates were scaled as described above. In the sensitivity analysis, we used weighted median, MR-Egger to examine the potential pleiotropy, and additionally employed RadialMR to account for the outlying SNPs (Additional file 1: Text S2). Similar to the description above, once the outlying SNPs were identified, we excluded them and repeated the above analyses.

All statistical analyses were conducted with R software (version 4.0.0 with packages, R Foundation for Statistical Computing, Vienna, Austria). Considering the bias due to multiple-testing, we used a Bonferroni-corrected threshold of *P* (two tailed) < 0.008 (0.05/6 outcomes) in the main analyses. We considered *P*-values (two tailed) between 0.008 and 0.05 as suggestive evidence of associations.

## Results

### Baseline characteristics

The final study sample included 335,727 individuals (mean age [SD]: 56.9 years [8.0], 46.2% male). The details of demographic characteristics are listed in Table 1. Compared with the participants with a normal sleep duration (7 or 8 h per day), those with either short sleep or long sleep duration were generally older and less likely to be employed and had a lower education level and a higher level of social deprivation (Townsend deprivation index). As for metabolic health, the participants sleeping too much (≥ 9 h per day) or too little (≤ 6 h per day) presented with a higher rate of MetS and its five components.

**Table 1:** Baseline characteristics of participants in the UK Biobank (n = 335,727)

	Habitual sleep duration (hours)						
	4	5	6	7 or 8	9	10	11
No. of participants	2795	13,928	63,087	230,721	20,115	4650	431
Demographics							
Age (years), mean (SD)	57.2 ± 7.6	57.3 ± 7.7	56.7 ± 7.8	56.7 ± 8.1	59.1 ± 7.8	59.1 ± 7.8	57.5 ± 8.2
Male (%)	1216 (43.5)	6109 (43.9)	30,412 (48.2)	107,029 (46.4)	8835 (43.9)	2131 (45.8)	190 (44.1)
University or college degree (%)	378 (13.5)	2748 (19.7)	17,803 (28.2)	76,579 (33.2)	4902 (24.4)	841 (18.1)	85 (19.7)
Current smoker (%)	514 (18.4)	1986 (14.3)	7321 (11.6)	21,165 (9.2)	2068 (10.3)	677 (14.6)	67 (15.5)
Current employed (%)	1138 (40.7)	7358 (52.8)	38,932 (61.7)	134,692 (58.4)	7030 (34.9)	1141 (24.5)	83(19.3)
Frequent drinker^a^ (%)	845 (30.2)	5228 (37.5)	27,656 (43.8)	108,307 (46.9)	8745 (43.5)	1775 (38.2)	128 (29.7)
Townsend deprivation index^b^, mean (SD)	– 0.1 ± 3.5	– 0.8 ± 3.3	– 1.4 ± 3.0	– 1.7 ± 2.8	– 1.6 ± 3.0	– 0.8 ± 3.3	– 0.7 ± 3.4
Body mass index (kg/m^2^), mean (SD)	28.9 ± 5.7	28.3 ± 5.2	27.8 ± 4.9	27.1 ± 4.6	27.8 ± 4.9	28.9 ± 5.6	28.7 ± 5.8
Outcomes							
MetS (%)	1159 (41.5)	5179 (37.2)	20,205 (32.0)	66,381 (28.8)	7642 (38.0)	2221 (47.8)	212 (49.2)
Components of MetS							
Central obesity (%)	1283 (45.9)	5681 (40.8)	22,679 (35.9)	72,522 (31.4)	7765 (38.6)	2269 (48.8)	214 (49.7)
High blood pressure (%)	1921 (68.7)	9718 (69.8)	42,188 (66.9)	150,652 (65.3)	14,222 (70.7)	3389 (72.9)	306 (71.0)
Dyslipidemia (%)	732 (26.2)	3245 (23.3)	12,992 (20.6)	43,848 (19.0)	4702 (23.4)	1390 (29.9)	143 (33.2)
Hypertriglyceridemia (%)	1586 (56.7)	7477 (53.7)	31,362 (49.7)	109,253 (47.4)	11,285 (56.1)	2911 (62.6)	276 (64.0)
Hyperglycemia (%)	345 (12.3)	1428 (10.3)	5111 (8.1)	15,939 (6.9)	2064 (10.3)	757 (16.3)	77 (17.9)

MetS: Metabolic syndrome; SD: Standard deviation

^a^Lower income was defined as average total household income before tax less than 18,000

^b^Townsend deprivation index was calculated based on the preceding national census output areas prior to participant joining UK Biobank. Each participant is assigned a score corresponding to their postcode location with a lower score indicating lower level of social deprivation

This table shows all the baseline covariates associated with sleep duration, with *P* < 0.001 for trend across different stratification

### Association between unweighted GRS of self-reported sleep duration and MetS

The demographic characteristics were compared between groups in different degrees of GRS of self-reported sleep duration (Additional file 1: Table S6). A lower GRS was significantly associated with a shorter sleep duration with *P* < 0.0001 for trend across categories. The prevalence of MetS (*P* for trend = 0.002) and two components including central obesity (*P* for trend < 0.0001) and hyperglycemia (*P* for trend = 0.0005) dramatically differed among participants with different degrees of GRS (Additional file 1: Table S7).

Taking the group with the lowest GRS as the reference, the associations between GRS and each metabolic outcome were examined (Table 2). Compared to the referenced group, the group with highest GRS showed 13% lower odds for MetS (*P* = 0.002), 18% lower odds for central obesity (*P* < 0.0001), and 16% lower odds for hyperglycemia (*P* = 0.0005).

**Table 2:** Associations between genetic risk score quartiles of sleep duration and metabolic outcomes in the UK Biobank (n = 335,727)

	GRS						
	Lowest GRS	Intermediate GRS		Highest GRS		*P* for trend	
		OR (95% CI)	*P* value	OR (95% CI)	*P* value	OR (95% CI)	*P* value
MetS	Ref	0.98 (0.97, 1.00)	0.118	0.97 (0.95, 0.99)	0.002	0.98 (0.97, 0.99)	0.002
MetS components							
Central obesity	Ref	0.96 (0.95, 0.98)	< 0.0001	0.92 (0.90, 0.94)	< 0.0001	0.96 (0.95, 0.97)	< 0.0001
High blood pressure	Ref	1.00 (0.98, 1.02)	0.937	1.01 (0.99, 1.03)	0.395	1.00 (0.99, 1.01)	0.404
Dyslipidemia	Ref	1.00 (0.98, 1.02)	0.985	0.99 (0.97, 1.01)	0.401	0.99 (0.98, 1.00)	0.412
Hypertriglyceridemia	Ref	0.98 (0.97, 1.00)	0.085	0.98 (0.96, 1.00)	0.078	0.99 (0.98, 1.00)	0.074
Hyperglycemia	Ref	0.98 (0.95, 1.01)	0.118	0.94 (0.90, 0.97)	0.0005	0.97 (0.95, 0.99)	0.0005

GRS: Genetic risk score; MetS: Metabolic syndrome

Each model was adjusted for age, sex, assessment centers, top 10 genetic PCs, genotyping array. Statistical significance was defined as Bonferroni-corrected threshold of *P* < 0.008 (0.05/6)

### Linear Mendelian randomization analyses of sleep duration with MetS

**Fig. 1:**
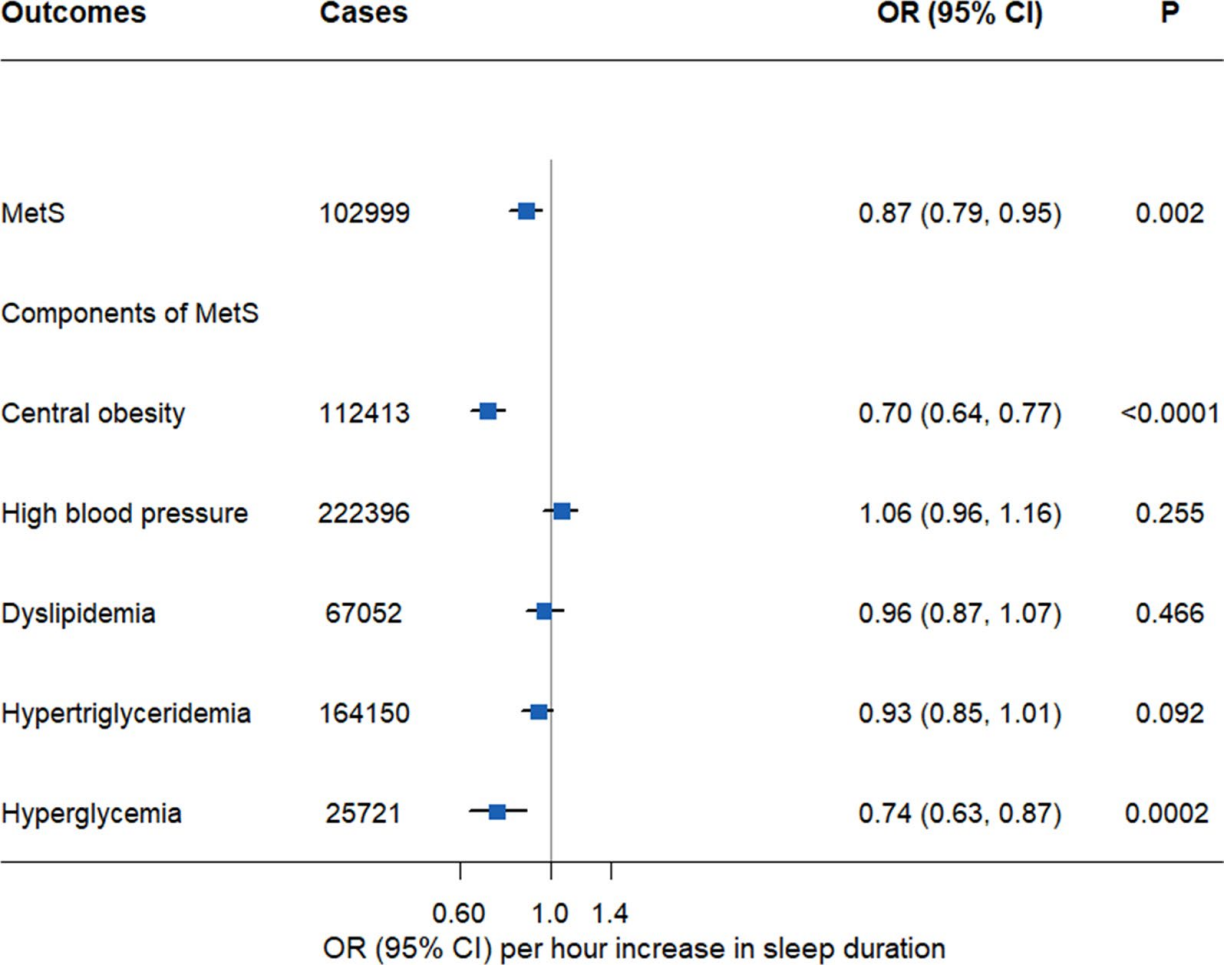
Linear Mendelian randomization estimates for the associations between genetically predicted sleep duration with MetS and its five components. CI: Confidence interval; MetS: Metabolic syndrome; OR: Odds ratios. The two-stage least squares regression models for each outcome were adjusted by age, sex, assessment center, top 10 genetic principal components and genotyping array. Statistical significance was defined as Bonferroni-corrected threshold of *P* < 0.008 (0.05/6)

The linear MR analyses demonstrated that genetically predicted 1 h longer sleep duration was significantly associated with 13% lower odds of MetS (adjusted OR = 0.87, 95% confidence interval [CI] 0.79–0.95), 30% lower odds of central obesity (adjusted OR = 0.70, 95% CI 0.64–0.77), and 26% lower odds of hyperglycemia (adjusted OR = 0.74, 95% CI 0.63–0.87), but not high blood pressure, dyslipidemia, or hypertriglyceridemia (Fig. 1). These patterns were similar with those found in the associations between unweighted GRS of self-reported sleep duration and metabolic outcomes (Table 2).

After adjusting for the additional potential confounders that were significantly associated with the GRS such as insomnia, napping, TDI, education, employed status, frequent drinking (Additional file 1: Table S8), the findings were consistent with those from main analyses. Moreover, after excluding the participants involved night-shift works, the results were still consistent (Additional file 1: Table S9). Next, by using MR-IVW method, 1-h increase in genetically predicted continuous sleep duration significantly predicted decreased risk of MetS (adjusted OR = 0.89, 95% CI 0.82–0.98), and the two components, namely, central obesity (adjusted OR = 0.74, 95% CI 0.68–0.80) and hyperglycemia (adjusted OR = 0.79, 95% CI 0.68–0.92) (Additional file 1: Table S10); the weighted median analyses demonstrated a similar trend of the causal associations. The intercepts of MR-Egger were not significantly different than zero, suggested no potential horizontal pleiotropy.

After excluding genetic outliers (Additional file 1: Fig. S3), the IVW analyses showed that genetically predicted one hour longer sleep duration was only associated with a 13% reduced risk of central obesity (adjusted OR = 0.87, 95% CI 0.80–0.96).

### Non-linear Mendelian randomization analyses of sleep duration with MetS

In the non-linear MR analyses, we found that genetically predicted sleep duration showed L-shaped associations with risk of MetS (Quadratic *P* < 0.0001; Cochran Q *P* < 0.0001) and most of its components including central obesity (Quadratic *P* < 0.0001; Cochran Q *P* < 0.0001), dyslipidemia (Quadratic *P* = 0.0001; Cochran Q *P* < 0.0001), hypertriglyceridemia (Quadratic *P* < 0.0001; Cochran Q *P* < 0.0001), and hyperglycemia (Quadratic *P* < 0.0001; Cochran Q *P* < 0.0001), except that the association between genetically predicted sleep duration and high blood pressure demonstrated as U-shaped (Quadratic *P* = 0.001; Cochran Q *P* = 0.0001) (Additional file 1: Fig. S4). The LACE estimates suggested genetically predicted sleep duration generated causally detrimental effects on MetS and most of its components in the short duration strata but not in the long one (Additional file 1: Fig. S4). The above findings implicated that it was more appropriate to use non-linear model rather than linear model in MR analyses to examine the associations between genetically predicted sleep duration and MetS.

### Associations of genetically predicted short and long sleep durations and MetS

**Fig. 2:**
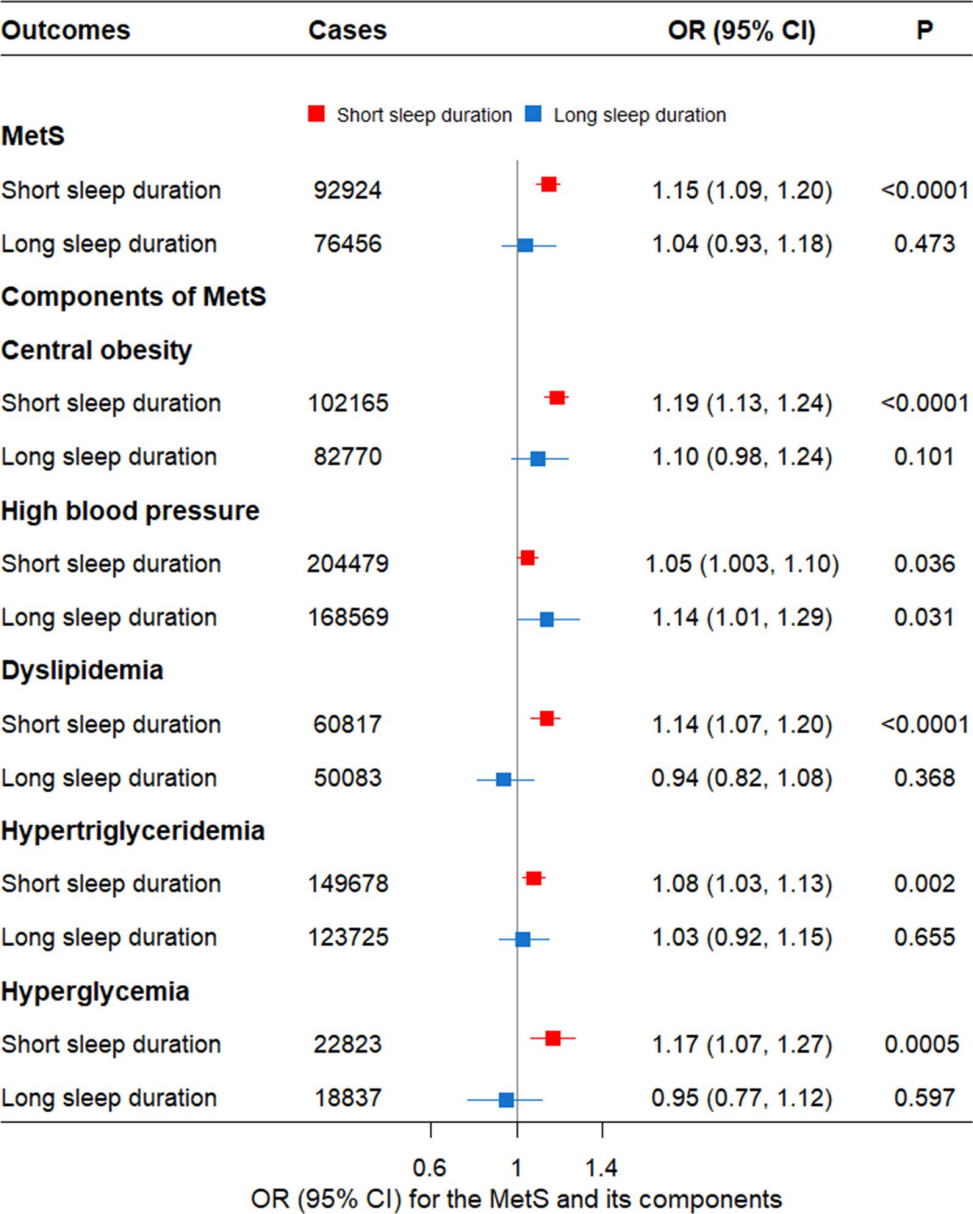
Mendelian randomization estimates for genetically predicted short and long sleep durations with MetS and its five components. CI: Confidence interval; MetS: Metabolic syndrome; OR: Odds ratios. Each mode was adjusted by age, sex, assessment center, top 10 genetic principal components and genotyping array. Statistical significance was defined as Bonferronif-corrected threshold of *P* < 0.008

In the complementary analyses, the genetically predicted short sleep duration was strongly associated with increased risk of MetS and almost all of its components including central obesity, dyslipidemia, hypertriglyceridemia, and hyperglycemia (*P* < 0.008). However, it only showed suggestive evidence that genetically predicted sleep duration caused high blood pressure (*P* = 0.036) (Fig. 2). Conversely, genetically predicted long sleep duration was not causal factor of MetS or any of its components (*P* > 0.008) (Fig. 2).

In the sensitivity analyses, almost all the IVW and weighted median estimates suggested the similar directions in both short and long sleep duration as those in the main analyses. The MR-Egger analyses showed absence of any pleiotropy (Additional file 1: Tables S11 and S12). After excluding genetic outliers of short sleep duration by Radial MR (Additional file 1: Fig. S5), the odds estimate of the causal association between genetically predicted short sleep duration and each metabolic outcome was almost the same as the main analyses. After excluding genetic outliers of long sleep duration by Radial MR (Additional file 1: Fig. S6), genetically predicted long sleep duration was not associated with any metabolic outcome.

## Discussion

Using linear MR method, we found that genetically predicted 1-h increase of sleep duration was associated with reduced risk of MetS and its two components (e.g., central obesity and hyperglycemia). We further confirmed non-linear rather than linear causal associations of genetically predicted sleep duration with MetS and all of its components. Our complementary analyses provided further evidence on the adverse effects of genetically predicted short sleep duration on the risks of MetS and its components, except for high blood pressure. Thus, our study provided evidence supporting that genetically predicted long sleep duration was unlikely to be a causal factor of most of the cardiometabolic traits. The findings suggest that linear assumption may be inappropriate to examine the association between sleep duration and MetS. This may explain why prior MR studies using linear method failed to find causal associations between overall continuous sleep duration and various metabolic traits like obesity [9, 14], serum lipids [16, 19], and impaired glycemic metabolism [15, 17, 18, 20].

Traditional observational studies have consistently found a strong relationship between short sleep duration and increased risk of MetS [5–8]. In line, our one-sample MR analyses demonstrated that genetically predicted short sleep duration was causally associated with almost all components of MetS including central obesity, dyslipidemia, hypertriglyceridemia, and hyperglycemia. However, in terms of single component, two recent two-sample MR studies found no evidence of causal role of genetically predicted short sleep duration on impaired glucose metabolism [18, 20]. The inconsistency may arise from the heterogenous definitions of each metabolic outcome. Bos et al., did not find a causal association between sleep duration and glycemic traits, including fasting glucose and fasting insulin [18]. However, the present study used HbA1c rather than fasting glucose to define hyperglycemia, and it is believed that fasting glucose only reflects short-term glucose levels. In contrast, HbA1c could reflect an average change in glucose levels over the past two to three months. Moreover, it was possible that the results of two-sample MR studies bias towards the null hypothesis due to weak instrument [35], for example, one of them leveraged only 17 SNPs to compute the genetically predicted short sleep duration [20], while the other MR studies and ours selected 27 SNPs [25, 36]. As suggested by experimental studies of short-term sleep deprivation, the mechanisms underlying the causal associations between short sleep duration and MetS may include hyperactivation of the hypothalamic–pituitary–adrenal axis and sympathetic nervous system, increased insulin resistance, and a boost of energy-intake behaviors regulated by appetite-regulating hormones (ghrelin, leptin, or endocannabinoids) as well as brain areas responsible for appetite and reward [11–13]. Our findings extend the current knowledge that long-term exposure to sleep loss does harm to metabolic health.

It has been long debated whether over-sleeping served as a casual risk factor or just an indicator of MetS [37, 38]. However, it is impractical and unethical to conduct long-term sleep extension experiment, particularly in those without sleep loss. The present study offers the first evidence supporting that habitual long sleep duration might not be a causal factor of MetS using linear and non-linear MR frameworks. These findings are also consistent with other MR studies that failed to show any causal links of long sleep duration with risks of other diseases, like cardiovascular diseases [25] and chronic kidney disease [36]. Taken together, our finding helps clarify the above debate by providing compelling evidence supporting the assertion that long sleep duration is potentially an indicator rather than a causal risk factor of MetS.

Our findings suggest that short rather than long sleep duration may be a potential causal risk factor for MetS and its components, such as central obesity and hyperglycemia. A recent experimental study confirmed that participants with sleep restriction for 4 h per day are predisposed to gain body weight and particularly for central accumulation of fat [39]. Moreover, a recent randomized clinical trial found that an extension of sleep duration 1.2 h per night would reduce the energy intake among adults with overweight who had habitual short sleep duration (less than 6.5 h per night) [40]. These results suggested that improving and maintaining healthy sleep duration is important to obesity prevention in adults, which is particularly related to cardiometabolic health. It has been demonstrated that a rigorously designed MR study can usually provide more reliable evidence to guide the development of RCT or provide information about potential public health when RCT (e.g., due to ethical reasons) cannot be implemented [41]. Therefore, our study may guide further studies' directions. It should be possible to conduct a randomized trial of sleep intervention for the prevention of MetS and its subsequent cardiometabolic disorders.

The major strength of this study is to use a MR design, which minimizes the potential biases due to confounding and reverse causality in the observational studies. Another strength is that non-linear MR methods were employed to delineate the shape of causal associations between sleep duration and MetS, which overcoming the limitations of prior MR studies that used linear methods found an absence of causal associations between continuous sleep duration and metabolic traits [16, 18]. Additionally, the inclusion of a large-scale study and multiple SNPs representing exposure risk enhances the statistical power to detect the association between genetically predicted sleep duration and MetS.

The findings of the current study should be, however, interpreted in the consideration of its limitations. First, sleep duration was assessed subjectively with a self-reported question, which may introduce measurement error. Second, MR estimation could be susceptible to horizontal pleiotropy, a phenomenon that genetic variants independently associate with observed traits other than the ones under investigation. The confounders associated with the genetic variants (e.g., Townsend deprivation index, and education level of each participant) may bias the main results. We additionally performed sensitivity analyses adjusting these confounders and found the conclusions of this study are not likely to be affected by horizontal pleiotropy. Third, one might concern that the results from one-sample MR analyses can be biased by weak instruments since a limited number of SNPs constituted genetically predicted long sleep duration. In this study, the estimated F-statistic over 10 for SNP-sleep duration led us to be confident to claim that the results were unlikely to be subject to significant bias due to weak instrument. Fourth, there are several genetic loci that directly overlapped with other GWAS signals, such as FTO and FADS1/2 gene clusters, which indicate the cardiometabolic risk. It has been suggested that the overlapped genetic loci may bring up the issue of potential pleiotropy in MR study [42]. However, we excluded some potential outlier SNPs in the sensitivity analysis, such as rs9940646, a marker in the FTO gene. The results were largely consistent with the main analysis. These results suggest that the pleiotropy effect caused by overlapped genetic loci shall be more minor if they have. Fifth, the lack of independent replication of our results may also be a potential limitation. However, two-sample MR method is not feasible in the current study due to the lack of available GWAS datasets for MetS and all of its components in the current literature. Despite a lack of replication, a recent study selecting the same 78 SNPs as ours has confirmed the association between the GRS of self-reported sleep duration and obesity [9]. In addition, most p values regarding the major findings in the current study were far below 0.008, which indicates a lower chance of type I error.

## Conclusions

This MR study offers evidence supporting a potential causal association between short sleep duration and MetS and four out of its five individual components. Our study also suggests that long sleep duration is unlikely to be a causal risk factor of cardiometabolic traits. Collectively, these findings suggest that sleep compensation for those with sleep loss probably contributes to alleviate the metabolic epidemic.

## Supplementary Information

**Additional file 1: Figure S1. **Flow chart of participant selection. **Figure S2.** Brief introduction of the assumptions of Mendelian randomization. **Text S1.** Association between the unweighted GRS of self-reported sleep duration and MetS in UK Biobank. **Text S2.** Introduction of MR inverse-variance weighted, MR weighted median and MR-Egger analyses. **Table S1.** Medications for high blood pressure, low HDL cholesterol, hypertriglyceridemia, and hyperglycemia. **Table S2.** 78 single-nucleotide polymorphisms and effect sizes for continuous sleep duration identified in UK Biobank. **Table S3.** 27 single-nucleotide polymorphisms and effect sizes for short sleep duration identified in UK Biobank. **Table S4.** 8 single-nucleotide polymorphisms and effect sizes for long sleep duration identified in UK Biobank. **Table S5.** Statistical power in the Mendelian randomization analyses of continuous sleep duration in relation to different outcomes per standard deviation (about 1 h) increase in sleep duration. **Table S6.** Baseline characteristics of participants stratified by the quartiles of genetic risk score in UK Biobank. **Table S7.** The number (percentages, %) of metabolic outcomes in each category of GRS group. **Table S8.** Associations between unweighted GRS and potential confounders in UK Biobank. **Table S9.** Sensitivity analyses of linear Mendelian randomization estimates for genetically predicted continuous sleep duration with adjustment for potential confounders. **Figure S3.** Radial Mendelian randomization plots for continuous sleep duration in metabolic outcomes. **Table S10.** Associations between genetically predicted 1-h increase in continuous sleep duration and metabolic outcomes using the inverse variance weighted, weighted median, and MR-Egger methods. **Figure S4.** Non-linear Mendelian randomization results between genetically predicted continuous sleep duration and metabolic outcomes using piecewise linear method. **Figure S5.** Radial Mendelian randomization plots for short sleep duration in metabolic outcomes. **Table S11.** Associations between genetically predicted short sleep duration and metabolic outcomes. **Figure S6.** Radial Mendelian randomization plots for long sleep duration in metabolic outcomes. **Table S12.** Associations between genetically predicted long sleep duration and metabolic outcomes.

## Abbreviations

CI: Confidence interval
GRS: Genetic risk score
HbA1c: Hemoglobin A1c
HDL: Low high-density lipoprotein
IVW: Inverse variance weighted
LACE: Localized average causal effect
MetS: Metabolic syndrome
MR: Mendelian randomization
OR: Odds ratios
RCT: Randomized controlled trial
SNPs: Single-nucleotide polymorphisms
TDI: Townsend deprivation index
