# Supplementary material for: Association between sleep duration and metabolic syndrome: linear and nonlinear Mendelian randomization analyses
Source: J Transl Med. 2023 Feb 7;21:90. doi: 10.1186/s12967-023-03920-2 (PMC9903442; doi:10.1186/s12967-023-03920-2)
Supplement: Supplementary file 1 — Additional file 1: Figure S1. Flow chart of participant selection. Figure S2. Brief introduction of the assumptions of Mendelian randomization. Text S1. Association between the unweighted GRS of self-reported sleep duration and MetS in UK Biobank. Text S2. Introduction of MR inverse-variance weighted, MR weighted median and MR-Egger analyses. Table S1. Medications for high blood pressure, low HDL cholesterol, hypertriglyceridemia, and hyperglycemia. Table S2. 78 single-nucleotide polymorphisms and effect sizes for continuous sleep duration identified in UK Biobank. Table S3. 27 single-nucleotide polymorphisms and effect sizes for short sleep duration identified in UK Biobank. Table S4. 8 single-nucleotide polymorphisms and effect sizes for long sleep duration identified in UK Biobank. Table S5. Statistical power in the Mendelian randomization analyses of continuous sleep duration in relation to different outcomes per standard deviation (about 1 h) increase in sleep duration. Table S6. Baseline characteristics of participants stratified by the quartiles of genetic risk score in UK Biobank. Table S7. The number (percentages, %) of metabolic outcomes in each category of GRS group. Table S8. Associations between unweighted GRS and potential confounders in UK Biobank. Table S9. Sensitivity analyses of linear Mendelian randomization estimates for genetically predicted continuous sleep duration with adjustment for potential confounders. Figure S3. Radial Mendelian randomization plots for continuous sleep duration in metabolic outcomes. Table S10. Associations between genetically predicted 1-h increase in continuous sleep duration and metabolic outcomes using the inverse variance weighted, weighted median, and MR-Egger methods. Figure S4. Non-linear Mendelian randomization results between genetically predicted continuous sleep duration and metabolic outcomes using piecewise linear method. Figure S5. Radial Mendelian randomization plots for short sleep duration in metabolic [file 12967_2023_3920_MOESM1_ESM.docx]

**Supplementary material online**

**Association between Sleep Duration and Metabolic Syndrome: Linear and Nonlinear Mendelian Randomization Analyses**

| **Figure S1** | Flow chart of participant selection. | Page 2 |
| --- | --- | --- |
| **Figure S2** | Brief introduction of the assumptions of Mendelian randomization. | Page 3 |
| **Text 1** | Association between the unweighted GRS of self-reported sleep duration and MetS in UK Biobank. | Page 4 |
| **Text 2** | Introduction of MR inverse-variance weighted, MR weighted median and MR-Egger analyses. | Page 4 |
| **Table S1** | Medications for high blood pressure, low HDL cholesterol, hypertriglyceridemia, and hyperglycemia. | Page 6 |
| **Table S2** | 78 single-nucleotide polymorphisms and effect sizes for continuous sleep duration identified in UK Biobank. | Page 7 |
| **Table S3** | 27 single-nucleotide polymorphisms and effect sizes for short sleep duration identified in UK Biobank. | Page 10 |
| **Table S4** | 8 single-nucleotide polymorphisms and effect sizes for long sleep duration identified in UK Biobank. | Page 11 |
| **Table S5** | Statistical power in the Mendelian randomization analyses of continuous sleep duration in relation to different outcomes per standard deviation (about 1 hour) increase in sleep duration. | Page 12 |
| **Table S6** | Baseline characteristics of participants stratified by the quartiles of genetic risk score in UK Biobank. | Page 13 |
| **Table S7** | The number (percentages, %) of metabolic outcomes in each category of GRS group. | Page 14 |
| **Table S8** | Associations between unweighted GRS and potential confounders in UK Biobank. | Page 15 |
| **Table S9** | Sensitivity analyses of linear Mendelian randomization estimates for genetically predicted continuous sleep duration with adjustment for potential confounders. | Page 16 |
| **Figure S3** | Radial Mendelian randomization plots for continuous sleep duration in metabolic outcomes | Page 17 |
| **Table S10** | Associations between genetically predicted one-hour increase in continuous sleep duration and metabolic outcomes using the inverse variance weighted, weighted median, and MR-Egger methods. | Page 18 |
| **Figure S4** | Non-linear Mendelian randomization results between genetically predicted continuous sleep duration and metabolic outcomes using piecewise linear method. | Page 19 |
| **Figure S5** | Radial Mendelian randomization plots for short sleep duration in metabolic outcomes. | Page 20 |
| **Table S11** | Associations between genetically predicted short sleep duration and metabolic outcomes. | Page 21 |
| **Figure S6** | Radial Mendelian randomization plots for long sleep duration in metabolic outcomes. | Page 22 |
| **Table S12** | Associations between genetically predicted long sleep duration and metabolic outcomes. | Page 23 |

**Figure S1. Flow chart of participant selection.**


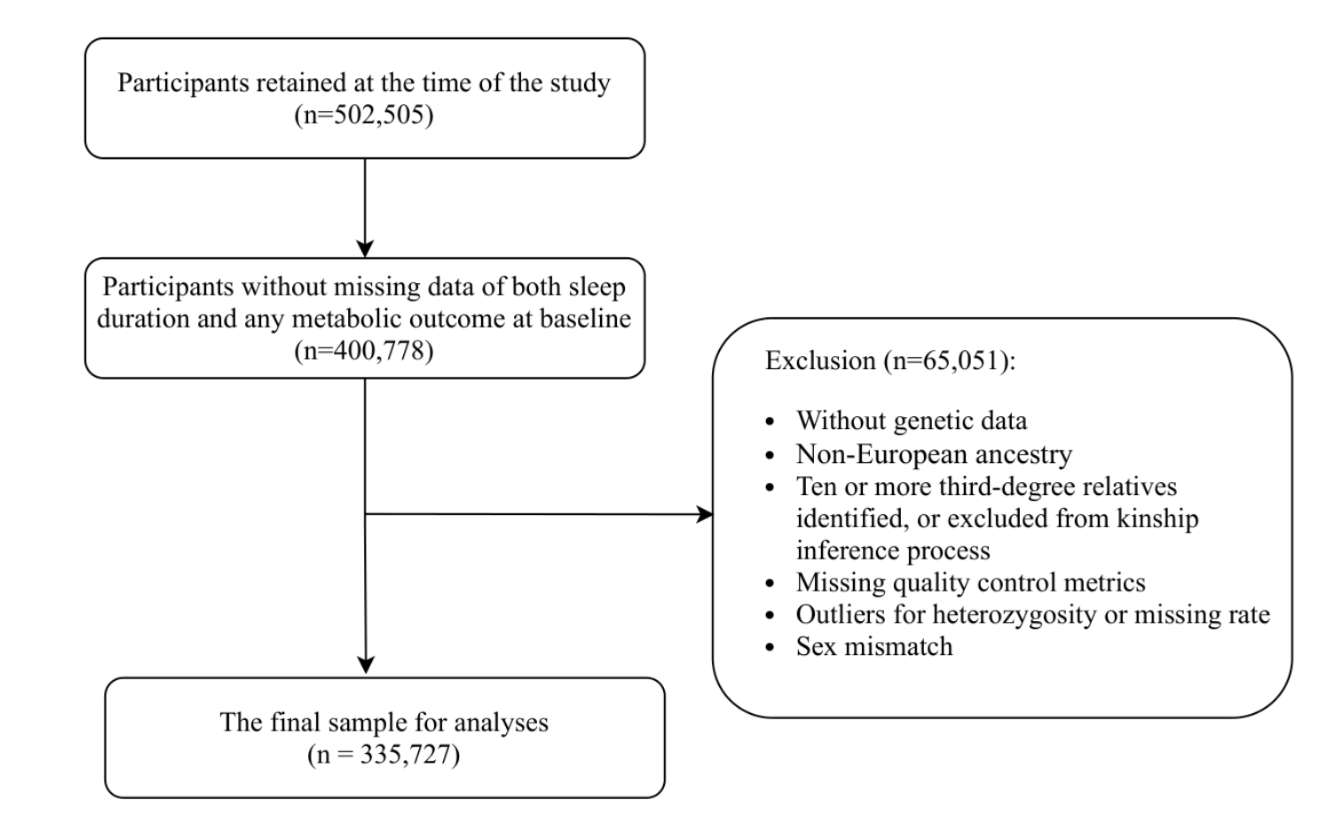


**Figure S2. Brief introduction of the assumptions of Mendelian randomization.**


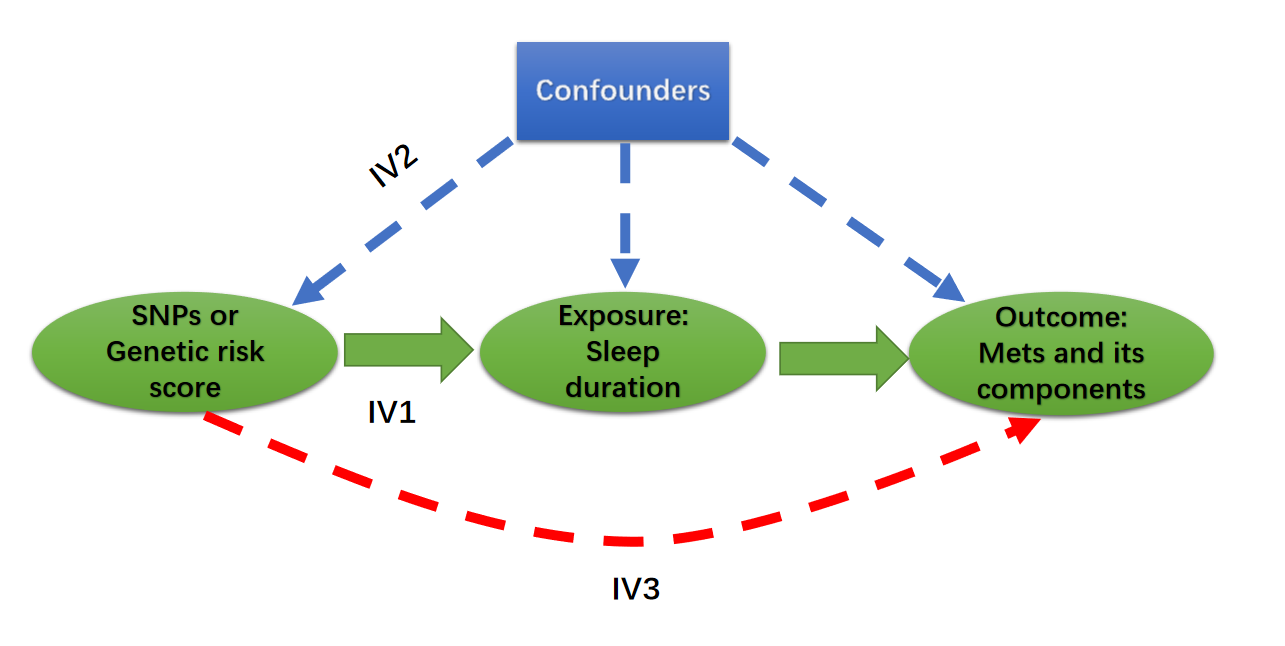


**Relevance assumption (IV1):** The genetic variants (SNPs or genetic risk scores of sleep duration) must be associated with the exposure or risk factor of interest (sleep duration).

**Independence assumption (IV2):** It should be lack of confounders confounding the association between genetic variant and the outcome (MetS and its components).

**Exclusion restriction (IV3):** The genetic variants (SNPs or genetic risk scores of sleep duration) cause the outcome must only via the corresponding exposure (sleep duration).

*Davies NM, Holmes MV, Davey Smith G. Reading Mendelian randomisation studies: a guide, glossary, and checklist for clinicians. BMJ. 2018 Jul 12;362:k601. doi: 10.1136/bmj.k601.*

**Text 1. Association between the unweighted GRS of self-reported sleep duration and MetS in UK Biobank.**

The methods used to investigate the association between the GRS of self-reported sleep duration and MetS in UK Biobank is as follow. As a natural randomization, the genetic alleles are fixed at conception and randomly assigned to individuals, therefore, we used the GRS generated from genetic alleles for grouping to mimic an RCT ^1^. Next, we categorized the participants into three groups based on their quartiles of GRS (Group 1: Q1; Group 2: Q2-3; Group 3: Q4). We then employed multivariate logistic or linear regression analyses to examine the associations between GRS of self-reported sleep duration and the metabolic outcomes, adjusting with age, sex, and other confounders as appropriate. Further, we excluded the participants with night-shift works and repeat the regression analyses.

**Text 2. Introduction of MR inverse-variance weighted, MR weighted median and MR-Egger analyses.**

The individual-level genetic data derived from UK Biobank imputation dataset were used to employ the MR analyses including inverse-variance weighted (IVW), weighted median and MR-Egger. First, the summarized data of SNP-exposure (self-reported sleep duration) and that of SNP-outcomes (the five components of MetS, such as central obsesity, hyperglycemia,etc) were derived by regressing each SNP against the exposure or outcome, respectively, adjusting with age, sex, assessment center, top 10 principal components of ancestry, and genotyping array, Second, we divided SNP-outcomes associations by SNP-exposure associations. Subsequently, we meta-analyzed across all SNPs in the genetic variants. For the sensitivity analyses, we used the “TwoSampleMR” package in a one sample dataset ^1^. First, the linkage disequilibrium clumping with a cutoff of R^2^ = 0.1 was adopted to guarantee the independence of the genetic variants for self-reported sleep duration ^2^. Second, the following methods were employed: fixed-effects IVW, random-effects IVW, weighted median, and MR Egger ^3,4^. In short, the fixed-effects IVW in a two-sample setting is the same with the weighted genetic risk score ^5^ in one-sample analyses. As for the random-effects IVW, it is appropriate to give valid estimate in the condition that horizontal pleiotropy exists or the pleiotropy is balanced to the null across all SNPs ^6^. Weighted median method can provide valid estimates even up to 50% of the information in the genetic variants to violate the assumption of no horizontal pleiotropy ^7^. MR-Egger method is used to estimate the effects of directional pleiotropy through using intercept of the model to infer the statistical significance of directional horizontal pleiotropy ^4^. Overall, the estimates of MR Egger and weighted median methods are less precise than that of the IVW method. Nevertheless, the conclusion will be more confident if the estimates from all the three methods are consistent ^6,8,9^. In addition, considering the bias caused by genetic outliers, we further applied Radial MR analyses to identify such outlier ^10^.

**References**

1. Hemani G, Zheng J, Elsworth B, et al. The MR-Base platform supports systematic causal inference across the human phenome. eLife. 2018; 7.

2. Daghlas I, Dashti HS, Lane J, et al. Sleep Duration and Myocardial Infarction. Journal of the American College of Cardiology. 2019; 74 (10): 1304-1314.

3. Bowden J, Davey Smith G, Haycock PC, Burgess S. Consistent Estimation in Mendelian Randomization with Some Invalid Instruments Using a Weighted Median Estimator. Genet Epidemiol. 2016; 40 (4): 304-314.

4. Bowden J, Davey Smith G, Burgess S. Mendelian randomization with invalid instruments: effect estimation and bias detection through Egger regression. International journal of epidemiology. 2015; 44 (2): 512-525.

5. Burgess S, Butterworth A, Thompson SG. Mendelian randomization analysis with multiple genetic variants using summarized data. Genet Epidemiol. 2013; 37 (7): 658-665.

6. Bowden J, Del Greco MF, Minelli C, Davey Smith G, Sheehan N, Thompson J. A framework for the investigation of pleiotropy in two-sample summary data Mendelian randomization. Statistics in medicine. 2017; 36 (11): 1783-1802.

7. Burgess S, Bowden J, Fall T, Ingelsson E, Thompson SG. Sensitivity Analyses for Robust Causal Inference from Mendelian Randomization Analyses with Multiple Genetic Variants. Epidemiology (Cambridge, Mass). 2017; 28 (1): 30-42.

8. Burgess S, Thompson SG. Interpreting findings from Mendelian randomization using the MR-Egger method. European journal of epidemiology. 2017; 32 (5): 377-389.

9. Davies NM, Holmes MV, Davey Smith G. Reading Mendelian randomisation studies: a guide, glossary, and checklist for clinicians. BMJ (Clinical research ed). 2018; 362: k601.

10. Bowden J, Spiller W, Del Greco MF, et al. Improving the visualization, interpretation and analysis of two-sample summary data Mendelian randomization via the Radial plot and Radial regression. International journal of epidemiology. 2018; 47 (4): 1264-1278.

**Table S1. Medications for high blood pressure, low HDL cholesterol, hypertriglyceridemia, and hyperglycemia.**

| **Outcomes** | **Medications** |
| --- | --- |
| High blood pressure | “Blood pressure medication” ( field ID 6177 or 6153). |
| Low HDL cholesterol | Nicotinic acid and derivatives: nicotinic acid product, niaspan 500mg m/r tablet, nicofuranose, nicotinyl alcohol, acipimox, olbetam 250mg capsule; Other lipid modifying agents: ezetimibe. |
| Hypertriglyceridemia | Statin: Simvastatin, velastatin, Pravastatin, eptastatin, Fluvastatin, lescol 20mg capsule, Atorvastatin, lipitor 10mg tablet, Rosuvastatin, crestor 10mg tablet; Fibrates: clofibrate, atromid-s 500mg capsule, bezafibrate, bezafibrate product, bezalip 200mg tablet, bezalip-mono 400mg m/r tablet, gemfibrozil, gemfibrozil product, lopid 300 capsule, fenofibrate, ciprofibrate. |
| Hyperglycemia | Insulin: insulin product; Sulphonylureas: glibenclamide, chlorpropamide, daonil 5mg tablet, semi-daonil 2.5mg tablet, diabetamide 2.5mg tablet, euglucon 2.5mg tablet, diabinese 100mg tablet, tolbutamide, glibornuride, glutril 25mg tablet, tolazamide, glibenese 5mg tablet, minodiab 2.5mg tablet, glipizide, glipizide product, gliquidone, glurenorm 30mg tablet, gliclazide, diamicron 80mg tablet, glimepiride, amaryl 1mg tablet, acetohexamide; Meglitinides: repaglinide, novonorm 0.5mg tablet, nateglinide, starlix 60mg tablet; Glitazones: troglitazone, romozin 200mg tablet, rosiglitazone, rosiglitazone 1mg / metformin 500mg tablet, pioglitazone, actos 15mg tablet; Others: glymidine, gondafon 500mg tablet, acarbose, glucobay 50mg tablet |

HDL, high-density lipoproteins.

**Table S2. 78 single-nucleotide polymorphisms and effect sizes for continuous sleep duration identified in UK Biobank.**

| SNP | Beta | SE | EA | NEA | EA_freq | P-value | Related gene(s) |
| --- | --- | --- | --- | --- | --- | --- | --- |
| rs10173260 | 0.77 | 0.1387932 | C | T | 0.606235 | 2.90E-08 | ***MAP2*** |
| rs10421649 | 0.798 | 0.1376772 | A | T | 0.55697 | 6.90E-09 | ***FBXL12****,* *UBL5, PIN1* |
| rs10483350 | 1.042 | 0.172056 | G | A | 0.195418 | 1.50E-09 | ***MIR548AI****, LOC100128215, PRKD1* |
| rs1057703 | 1.164 | 0.1923966 | G | T | 0.146608 | 1.10E-09 | ***BSX****, C11orf63* |
| rs10761674 | 0.74 | 0.1359546 | C | T | 0.477334 | 4.20E-08 | ***EGR2****, ADO* |
| rs10973207 | 1.226 | 0.1874364 | T | G | 0.157677 | 6.00E-11 | ***EBLN3****, ZCCHC7* |
| rs11190970 | 0.923 | 0.1693908 | G | A | 0.798661 | 4.60E-08 | ***BTRC****, FGF8, NFKB2, PITX3, PSD, FBXW4, GBF1, LDB1, NOLC1, NPM3, MGEA5, PPRC1 POLL, KCNIP2 CUEDC2, FBXL15, C10ORF76, HPS6, C10orf95, ELOVL3* |
| rs112230981 | 1.892 | 0.3136722 | A | G | 0.94984 | 2.20E-09 | ***ERC2*** |
| rs113113059 | 0.968 | 0.1642392 | T | C | 0.78 | 8.40E-09 | ***CUL9****, MEA1, PPP2R5D, SMAD5-AS2, SRF, CUL7, DNPH1, MRPL2, TTBK1, RRP36, KLC4, KLHDC3* |
| rs11567976 | 0.768 | 0.1371096 | T | C | 0.570908 | 2.10E-08 | ***CDC25C****, EGR1, ETF1, GFRA3, HSPA9, NME5, CDC23, KIF20A, BRD8, FAM53C, REEP2, KDM3B* |
| rs11602180 | 1.095 | 0.183948 | C | T | 0.836621 | 2.30E-09 | ***PTPRJ,*** *OR4X2, OR4B1, OR4S1, OR4X1* |
| rs11614986 | 0.983 | 0.1770606 | A | G | 0.820952 | 2.70E-08 | ***MMAB****, MVK, KCTD10, UBE3B, MYO1H* |
| rs11621908 | 1.446 | 0.2497788 | C | T | 0.917141 | 5.60E-09 | ***ADCK1,*** *FRDAP* |
| rs11643715 | 0.834 | 0.1497948 | G | C | 0.290942 | 3.20E-08 | ***PRKCB*** |
| rs11885663 | 0.973 | 0.1570866 | T | C | 0.247809 | 8.60E-10 | ***SCN1A****, GALNT3, TTC21B* |
| rs12246842 | 0.804 | 0.136455 | A | G | 0.459815 | 3.90E-09 | ***MLLT10****, DNAJC1, SKIDA1* |
| rs12567114 | 0.89 | 0.152418 | A | G | 0.275802 | 4.30E-09 | ***DPYD*** |
| rs12607679 | 1.208 | 0.1555704 | T | C | 0.737717 | 8.30E-15 | ***TCF4*** |
| rs12611523 | 0.758 | 0.1365762 | A | G | 0.545244 | 3.10E-08 | ***SPOPL*** |
| rs1263056 | 0.768 | 0.1366332 | A | G | 0.519099 | 2.00E-08 | ***BUD13*** |
| rs12791153 | 1.413 | 0.253014 | T | A | 0.081089 | 1.90E-08 | *LOC729790, LOC646195* |
| rs13088093 | 0.976 | 0.144141 | G | T | 0.336317 | 7.00E-12 | ***PPP2R3A****, PCCB, STAG1, MSL2* |
| rs13109404 | 1.872 | 0.2644974 | T | G | 0.928024 | 1.40E-12 | ***BANK1*** |
| rs151014368 | 0.966 | 0.1691748 | A | G | 0.206258 | 9.10E-09 | ***LMAN2****, FGFR4, SLC34A1, RGS14, PRELID1, NSD1, MXD3* |
| rs1517572 | 0.879 | 0.1376802 | C | A | 0.580536 | 1.50E-10 | *METT5D1, OR2BH1P* |
| rs1553132 | 0.87 | 0.1550682 | G | A | 0.258433 | 2.50E-08 | ***GRM5*** |
| rs17427571 | 0.83 | 0.1461264 | A | G | 0.684313 | 1.30E-08 | ***PRKG2*** |
| rs174560 | 0.815 | 0.146205 | C | T | 0.314215 | 2.80E-08 | ***FADS1****, FADS3, RAB3IL1, FADS2, MYRF, V27 TMEM258, FEN1* |
| rs17732997 | 0.776 | 0.137292 | C | G | 0.569098 | 1.20E-08 | ***FOXP1****, LOC100128160* |
| rs1776776 | 1.198 | 0.2046426 | T | C | 0.873832 | 4.90E-09 | ***ARRDC1****, MRPL41, EHMT1, ARRDC1-AS1, DPH7, ZMYND19* |
| rs180769 | 0.763 | 0.1376304 | T | C | 0.424698 | 2.30E-08 | ***TRPC7****, SMAD5, SMAD5-AS1* |
| rs1939455 | 1.226 | 0.2136708 | G | T | 0.879446 | 1.20E-08 | ***TRPC6*** |
| rs1991556 | 0.994 | 0.1634238 | G | A | 0.773765 | 1.00E-09 | ***MAPT****, STH, KANSL1,* *LOC644157, LOC644172* |
| rs205024 | 0.83 | 0.1396266 | T | C | 0.383735 | 3.90E-09 | ***SHISA6*** |
| rs2072727 | 0.795 | 0.1371036 | T | C | 0.43617 | 7.90E-09 | ***YWHAB****, PABPC1L* |
| rs2079070 | 1.053 | 0.1539828 | C | G | 0.264613 | 7.50E-12 | ***FOXP2*** |
| rs2139261 | 1.122 | 0.1738686 | G | C | 0.748584 | 8.50E-11 | ***KCNJ12*** |
| rs2192528 | 0.802 | 0.136152 | A | G | 0.480065 | 2.70E-09 | *LCORL, LOC645174* |
| rs2231265 | 0.897 | 0.1619502 | G | A | 0.772289 | 2.70E-08 | ***PNRC1*** |
| rs269054 | 0.819 | 0.1375962 | A | T | 0.422076 | 2.10E-09 | ***DAB1*** |
| rs3095508 | 0.921 | 0.1382622 | C | A | 0.593529 | 3.10E-11 | ***RBFOX1*** |
| rs330088 | 0.868 | 0.1366068 | C | T | 0.547012 | 2.70E-10 | ***PPP1R3B****, LOC100129150* |
| rs34354917 | 0.825 | 0.1500486 | C | A | 0.710472 | 3.90E-08 | ***ALG10B****, CPNE8* |
| rs34556183 | 1.015 | 0.1513938 | A | G | 0.719606 | 2.30E-11 | ***ZBED9****,* *SCAND3, LOC646160,* |
| rs34731055 | 1.168 | 0.1768668 | T | C | 0.18089 | 3.70E-11 | ***MAD1L1*** |
| rs35531607 | 0.77 | 0.1363668 | C | T | 0.474083 | 1.50E-08 | ***CCSER1*** |
| rs365663 | 0.878 | 0.1367142 | A | G | 0.545963 | 1.00E-10 | ***SLC6A3*** |
| rs374153 | 1.057 | 0.1861848 | C | T | 0.158085 | 9.10E-09 | ***SLC8A1*** |
| rs4128364 | 0.876 | 0.1434516 | C | T | 0.339025 | 1.40E-09 | *PABPCP2, LOC100133235* |
| rs4538155 | 0.779 | 0.1424298 | T | C | 0.647426 | 3.60E-08 | ***NR4A2,*** *GPD2* |
| rs4592416 | 0.881 | 0.136206 | G | A | 0.464407 | 9.30E-11 | ***HSD17B12*** |
| rs460692 | 1.263 | 0.1998984 | C | T | 0.137484 | 3.60E-10 | ***LINC01377****, LOC100132531, LOC285577* |
| rs4767550 | 0.858 | 0.138576 | G | A | 0.414138 | 6.30E-10 | ***KSR2*** |
| rs55658675 | 0.788 | 0.1421352 | C | T | 0.644938 | 2.00E-08 | ***MAX****, FNTB, GPX2, CHURC1, RAB15* |
| rs56372231 | 1.017 | 0.1439886 | T | C | 0.334093 | 2.20E-12 | ***PAM****, PPIP5K2, GIN1, C5ORF30* |
| rs61796569 | 0.927 | 0.1538658 | T | C | 0.269583 | 1.50E-09 | ***PDE4B*** |
| rs61985058 | 1.116 | 0.1937358 | T | C | 0.143176 | 1.30E-08 | ***RTN1*** |
| rs62120041 | 1.567 | 0.2744982 | T | C | 0.933902 | 9.60E-09 | ***MBOAT2*** |
| rs6575005 | 0.934 | 0.1585032 | T | C | 0.757854 | 4.40E-09 | ***NOVA1*** |
| rs7115226 | 1.594 | 0.2614794 | A | C | 0.073525 | 1.70E-09 | ***DRD2*** |
| rs72804080 | 1.068 | 0.1917852 | G | A | 0.149928 | 2.90E-08 | ***LINC01122****, LOC101927285* |
| rs73219758 | 0.984 | 0.1497156 | G | A | 0.708064 | 5.60E-11 | ***SGCZ*** |
| rs7503199 | 0.885 | 0.1538298 | C | T | 0.734267 | 1.00E-08 | ***PER1****, PFAS, SMAD5-AS5, VAMP2, AURKB, ARHGEF15, RANGRF, BORCS6, CTC1, TMEM107, KRBA2, SLC25A35* |
| rs75539574 | 2.175 | 0.2439132 | C | A | 0.085792 | 6.90E-19 | ***VRK2****, LINC01122* |
| rs7556815 | 2.443 | 0.1644138 | A | G | 0.219144 | 1.30E-49 | ***PAX8****, LOC100130100* |
| rs7616632 | 0.792 | 0.1361532 | T | G | 0.522135 | 4.30E-09 | *IL20RB, NPM1P17* |
| rs7644809 | 0.784 | 0.1380888 | T | C | 0.421606 | 1.60E-08 | ***BBX****, LOC285205* |
| rs7806045 | 0.887 | 0.1575462 | T | C | 0.754703 | 1.40E-08 | ***CHCHD3*** |
| rs7915425 | 1.144 | 0.1793754 | T | C | 0.174682 | 2.00E-10 | ***BUB3****, LOC100131719, GPR26* |
| rs7951019 | 2.213 | 0.391251 | G | T | 0.032227 | 1.20E-08 | ***KMT2A****,* *ARCN1, IFT46, TMEM25* |
| rs80193650 | 1.01 | 0.1840338 | G | A | 0.162466 | 4.10E-08 | ***ZBTB9****, KIFC1, PHF1, SYNGAP1, CUTA* |
| rs8038326 | 0.955 | 0.152442 | A | G | 0.72691 | 2.80E-10 | ***SEMA6D*** |
| rs8050478 | 0.96 | 0.1358856 | G | A | 0.500253 | 1.70E-12 | ***GNAO1*** |
| rs915416 | 1.156 | 0.1496712 | C | G | 0.289947 | 9.90E-15 | ***CSMD2****, C1orf94, GJB5* |
| rs9345234 | 0.781 | 0.1379358 | C | A | 0.578016 | 1.80E-08 | *LOC100129847, LOC100128159* |
| rs9382445 | 0.872 | 0.1400322 | T | C | 0.62305 | 4.80E-10 | ***FAM83B*** |
| rs9903973 | 0.766 | 0.1363164 | C | T | 0.46702 | 2.60E-08 | *CA10, LOC339209* |
| rs9940646 | 1.017 | 0.1374474 | C | G | 0.577569 | 1.20E-13 | ***FTO*** |

SNP, single-nucleotide polymorphisms; SE, standard error; EA, effect allele; NEA, non-effect allele; EA_freq, effect allele frequency.

**Table S3. 27 single-nucleotide polymorphisms and effect sizes for short sleep duration identified in UK Biobank.**

| SNP | Beta | SE | EA | NEA | EA_freq | *P*-value | Related gene(s) |
| --- | --- | --- | --- | --- | --- | --- | --- |
| rs11763750 | 0.035 | 0.007941 | G | A | 0.814346 | 5.10E-09 | ***MAD1L1*** |
| rs1229762 | 0.037 | 0.00643 | T | C | 0.664501 | 1.00E-12 | ***FOXP2*** |
| rs12518468 | 0.031 | 0.006468 | C | T | 0.328456 | 8.50E-09 | ***ADCY2****, POLS, LOC100130063* |
| rs12567114 | 0.036 | 0.006436 | G | A | 0.7246 | 4.10E-09 | ***DPYD*** |
| rs12661667 | 0.028 | 0.006493 | T | C | 0.263495 | 2.80E-08 | ***USP49*** |
| rs12963463 | 0.029 | 0.006487 | C | T | 0.299425 | 1.90E-11 | ***TCF4*** |
| rs13107325 | 0.075 | 0.011003 | T | C | 0.074528 | 2.50E-13 | ***SLC39A8*** |
| rs1380703 | 0.035 | 0.005944 | G | A | 0.383531 | 1.60E-11 | ***VRK2****, LOC647016, LOC100131953* |
| rs142180737 | 0.154 | 0.03156 | C | T | 0.009491 | 4.40E-09 | ***ZSCAN12****, ZSCAN31, ZKSCAN3* |
| rs1607227 | 0.031 | 0.006474 | G | T | 0.704938 | 1.50E-09 | *METT5D1, OR2BH1P* |
| rs17005118 | 0.03 | 0.00648 | A | G | 0.264936 | 2.50E-09 | ***RASGEF1B****, PRKG2* |
| rs17388803 | 0.053 | 0.009774 | C | A | 0.105648 | 6.50E-10 | ***SEMA6D*** |
| rs2014830 | 0.03 | 0.00648 | C | T | 0.698128 | 2.70E-08 | ***RBM5****, AMT, APEH, RHOA, DAG1, GNAI2, GNAT1, GPX1, HYAL1, MST1, MST1R, SEMA3F, TCTA, UBA7 USP4, IFRD2, SEMA3B, HYAL3 HYAL2, BSN, CACNA2D2, IP6K1, RBM6, TRAIP, NPRL2, SLC38A3, CYB561D2, TMEM115, RASSF1, TUSC2, NAT6, GMPPB, ZMYND10, RNF123, CAMKV, NICN1, MON1A, LSMEM2, AMIGO3 FAM212A, ACTBP13* |
| rs205024 | 0.031 | 0.006474 | C | T | 0.616724 | 2.70E-08 | ***SHISA6*** |
| rs2186122 | 0.024 | 0.006014 | T | A | 0.561566 | 4.80E-09 | ***PDE4B*** |
| rs2820313 | 0.031 | 0.005973 | G | A | 0.341112 | 2.30E-09 | ***LMOD1****, IPO9, NAV1, SHISA4* |
| rs2863957 | 0.054 | 0.007299 | C | A | 0.781508 | 2.60E-18 | ***PAX8****, LOC100130100* |
| rs3776864 | 0.031 | 0.006468 | A | C | 0.66721 | 1.70E-08 | ***PAM****, PPIP5K2, GIN1, c5orf30* |
| rs4585442 | 0.031 | 0.005973 | G | A | 0.311023 | 8.10E-10 | ***SMAD5****, SMAD5-AS1, TRPC7* |
| rs5757675 | 0.034 | 0.006948 | G | T | 0.259528 | 2.70E-09 | ***MGAT3****, SYNGR1, TAB1* |
| rs59779556 | 0.025 | 0.006008 | T | G | 0.553827 | 2.00E-08 | ***GNAO1****, AMFR* |
| rs60882754 | 0.055 | 0.012212 | A | T | 0.938985 | 1.80E-08 | ***PCMTD1****, PXDNL* |
| rs7524118 | 0.03 | 0.00648 | C | T | 0.708376 | 4.90E-08 | ***CSMD2****, C1orf94, GJB5* |
| rs75539574 | 0.045 | 0.010845 | A | C | 0.914664 | 8.40E-11 | ***LINC01122*** |
| rs7939345 | 0.035 | 0.007441 | T | G | 0.207569 | 4.00E-08 | ***PTPRJ****, NDUFS3, PSMC3, RAPSN, CELF1, NUP160, FNBP4, MTCH2, KBTBD4, AGBL2, C1QTNF4* |
| rs9321171 | 0.031 | 0.005967 | C | T | 0.540122 | 4.20E-08 | ***LAMA2****, ARHGAP18* |
| rs9367621 | 0.024 | 0.00551 | T | A | 0.43104 | 1.60E-08 | ***HCRTR2****, GFRAL* |

SNP, single-nucleotide polymorphisms; SE, standard error; EA, effect allele; NEA, non-effect allele; EA_freq, effect allele frequency.

**Table S4. 8 single-nucleotide polymorphisms and effect sizes for long sleep duration identified in UK Biobank.**

| SNP | Beta | SE | EA | NEA | EA_freq | *P*-value | Related gene(s) |
| --- | --- | --- | --- | --- | --- | --- | --- |
| rs10899257 | 0.067659 | 0.01304 | A | G | 0.144473 | 4.60E-08 | *GUCY2E* |
| rs17688916 | 0.07139 | 0.012503 | T | A | 0.796267 | 1.10E-11 | *KIAA1267, LOC644246* |
| rs17817288 | 0.039221 | 0.009407 | A | G | 0.518127 | 8.90E-09 | *FTO* |
| rs3751046 | 0.069526 | 0.013503 | G | A | 0.147342 | 2.00E-08 | *C11orf63* |
| rs549961083 | 0.533565 | 0.116474 | T | C | 0.001432 | 9.60E-09 | *RAB3C, PDE4D* |
| rs6737318 | 0.076035 | 0.010993 | G | A | 0.221841 | 3.40E-13 | *PAX8, LOC100130100* |
| rs7534398 | 0.046884 | 0.01182 | A | T | 0.201382 | 2.10E-08 | *CAMTA1* |
| rs75458655 | 0.184818 | 0.029237 | T | C | 0.022973 | 5.40E-12 | *MPZL2, JAML, MPZL3* |

SNP, single-nucleotide polymorphisms; SE, standard error; EA, effect allele; NEA, non-effect allele; EA_freq, effect allele frequency.

**Table S5. Statistical power in the Mendelian randomization analyses of continuous sleep duration in relation to different outcomes per standard deviation (about 1 hour) increase in sleep duration (n=335,727).**

| **Outcomes** | **Number**  **of cases** | **Power** |
| --- | --- | --- |
| **MetS** | 102,999 | 0.767 |
| ***Components of MetS*** |  |  |
| Central obesity | 112,413 | 1.000 |
| High blood pressure | 222,396 | 0.255 |
| Dyslipidemia | 67,052 | 0.103 |
| Hypertriglyceridemia | 164,150 | 0.342 |
| Hyperglycemia | 25,721 | 0.942 |

Online tool was used to calculate the MR power of each outcome (https://sb452.shinyapps.io/power/)

**Table S6. Baseline characteristics of participants stratified by the quartiles of genetic risk score in UK Biobank (N = 335,727).**

|  | **Genetic risk score** | | | **P for trend** |
| --- | --- | --- | --- | --- |
|  | **Lowest GRS**  **(< 25%)** | **Intermediate GRS**  **(25% to 75%)** | **Highest GRS**  **(> 75%)** |  |
| **Demographics** |  |  |  |  |
| No. of participants | 82,365 | 178,105 | 75,257 |  |
| Age (years) | 56.9 ± 8.00 | 56.9 ± 7.99 | 56.9 ± 8.03 | 0.844 |
| Male (%) | 38348 (46.6%) | 82573 (46.4%) | 35001(46.5%) | 0.813 |
| **Sleep duration (hours)** | 7.06 ± 1.04 | 7.17 ± 1.04 | 7.27 ± 1.04 | **<0.0001** |
| **Short sleep duration (%)** | 22351 (27.1%) | 42138 (23.7%) | 15321 (20.4%) | **<0.0001** |
| **Normal sleep duration (%)** | 54926 (66.7%) | 122670 (68.8%) | 53125 (70.6%) | **<0.0001** |
| **Long sleep duration (%)** | 5088 (6.2%) | 13297 (7.5%) | 6811 (9.0%) | **<0.0001** |
| **University or college degree (%)** | 24940 (30.3%) | 54615 (30.7%) | 23781 (31.6%) | **<0.0001** |
| Current smoker (%) | 8345 (10.1%) | 17845 (10.0%) | 7608 (10.1%) | 0.111 |
| **Frequent drinker ^a^ (%)** | 37189 (45.2%) | 80849 (45.4%) | 34646 (46.0%) | **0.0007** |
| **Current employed (%)** | 46957 (57.0%) | 100891 (56.6%) | 42526 (56.5%) | **0.025** |
| **Townsend deprivation index ^b^** | -1.54 ± 2.93 | -1.59 ± 2.92 | -1.61 ± 2.89 | **<0.0001** |

All values are indicated in means ± standard deviation (SD) or n (percentages, %). ^a^ Frequent drinker was defined as drinking two or more times per week; ^b^ Townsend deprivation index was calculated based on the preceding national census output areas prior to participant joining UK Biobank. Each participant is assigned a score corresponding to their postcode location; a lower score represents lower deprivation.

**Table S7. The number (percentages, %) of metabolic outcomes in each category of GRS group (N = 335,727).**

| **Outcomes** | **Lowest GRS**  **(n = 82,365)** | **Intermediate GRS**  **(n = 178,105)** | **Highest GRS**  **(n = 75,257)** | ***P* for trend*** |
| --- | --- | --- | --- | --- |
| **MetS** (%) | 25518 (31.0) | 54680 (30.7) | 22801 (30.3) | **0.002** |
| **Components of MetS** |  |  |  |  |
| **Central obesity (%)** | 28249 (34.3) | 59653 (33.5) | 24511 (32.6) | **<0.0001** |
| Higher blood pressure (%) | 54517 (66.2) | 117937 (66.2) | 49942 (66.4) | 0.403 |
| Dyslipidemia (%) | 16480 (20.0) | 35639 (20.0) | 14933 (19.8) | 0.412 |
| Hypertriglyceridemia (%) | 40500 (49.2) | 86953 (48.8) | 36697 (48.8) | 0.0839 |
| **Hyperglycemia (%)** | 6467 (7.8) | 13686 (7.7) | 5568 (7.4) | **0.0005** |

*Adjusted for age and sex. Statistical significance was defined as Bonferroni-corrected threshold of *P* < 8.3E-3 (0.5/6).

**Table S8. Associations between unweighted GRS and potential confounders in UK Biobank (N = 335,727).**

| **Instrument** | **Confounder** | **Beta** | **SE** | **N** | ***P* value** |
| --- | --- | --- | --- | --- | --- |
| Sleep duration scores | **Insomnia** | -0.19155 | 0.01325 | 335518 | **<0.0001*** |
| Sleep duration scores | **Napping** | 0.18165 | 0.01605 | 335638 | **<0.0001*** |
| Sleep duration scores | **TDI** | -0.014910 | 0.003273 | 335323 | **<0.0001*** |
| Sleep duration scores | **Education** | 0.10381 | 0.02070 | 332986 | **<0.0001*** |
| Sleep duration scores | **Employ status** | -0.05045 | 0.01929 | 334833 | **0.008** |
| Sleep duration scores | **Frequent drinker** | -0.026082 | 0.006432 | 335726 | **<0.0001*** |
| Sleep duration scores | Daytime sleepiness | -0.03629 | 0.01952 | 334762 | 0.063 |
| Sleep duration scores | Age | 1.858E-04 | 1.192e-03 | 335726 | 0.876 |
| Sleep duration scores | Sex | -0.02117 | 0.01913 | 335726 | 0.142 |
| Sleep duration scores | Current smoker | -0.02083 | 0.01426 | 334628 | 0.144 |
| Sleep duration scores | Chronotype | 0.01201 | 0.01089 | 300555 | 0.270 |
| Sleep duration scores | Snoring | -0.005375 | 0.020387 | 313369 | 0.792 |

Coefficients are in terms of an average-SNP increase in the allele score per unit/level increase in confounder. SE, standard error; TDI, Townsend deprivation index,*associations surpassing multiple-testing correct *P*-value threshold of 0.05/12 = 0.004.

**Table S9.** **Sensitivity analyses of linear Mendelian randomization estimates for genetically predicted continuous sleep duration with adjustment for potential confounders.**

| **Outcomes** | **Cases** | **Model 1*** | | | **Model 2 ^#^** | | | **Cases^§^** | **Model 3 ^§^** | | |
| --- | --- | --- | --- | --- | --- | --- | --- | --- | --- | --- | --- |
|  |  | **OR** | **95% CI** | **P value** | **OR** | **95% CI** | **P value** |  | **OR** | **95% CI** | **P value** |
| **MetS** | 102,999 | **0.90** | **(0.82, 0.99)** | 0.030 | **0.88** | **(0.80, 0.97)** | **0.008** | 98,157 | **0.87** | **(0.79, 0.96)** | **0.004** |
| **Components of MetS** |  |  |  |  |  |  |  |  |  |  |  |
| **Central obesity** | 112,413 | **0.72** | **(0.67, 0.79)** | **<0.0001** | **0.71** | **(0.65, 0.78)** | **<0.0001** | 106,93 | **0.70** | **(0.64, 0.77)** | **<0.0001** |
| Higher blood pressure | 222,396 | 1.07 | (0.97, 1.18) | 0.137 | 1.07 | (0.98, 1.18) | 0.133 | 212,603 | 1.05 | (0.96, 1.16) | 0.276 |
| Dyslipidemia | 67,052 | 1.01 | (0.91, 1.12) | 0.850 | 0.98 | (0.88, 1.09) | 0.706 | 63,582 | 0.98 | (0.88, 1.09) | 0.713 |
| Hypertriglyceridemia | 164,150 | 0.95 | (0.87, 1.04) | 0.262 | 0.94 | (0.86, 1.03) | 0.195 | 156,331 | 0.92 | (0.84, 1.00) | 0.057 |
| **Hyperglycemia** | 25,721 | **0.78** | **(0.67, 0.92)** | **0.003** | **0.75** | **(0.64, 0.88)** | **<0.0001** | 24,672 | **0.75** | **(0.64, 0.88)** | **0.0005** |

*Adjusted for age, sex, assessment centers, top 10 genetic PCs, genotyping array, education, employment status, Townsend deprivation index, and frequent drinker.

**^#^**Additionally adjusted for other sleep confounders, such as insomnia and napping.

**^§^** Subgroup analyses by excluding participants involved in night-shift work.

Multiple-testing correct *P*-value threshold of 0.05/6 = 0.008.

**Figure S3.** **Radial Mendelian randomization plots for continuous sleep duration in metabolic outcomes.**


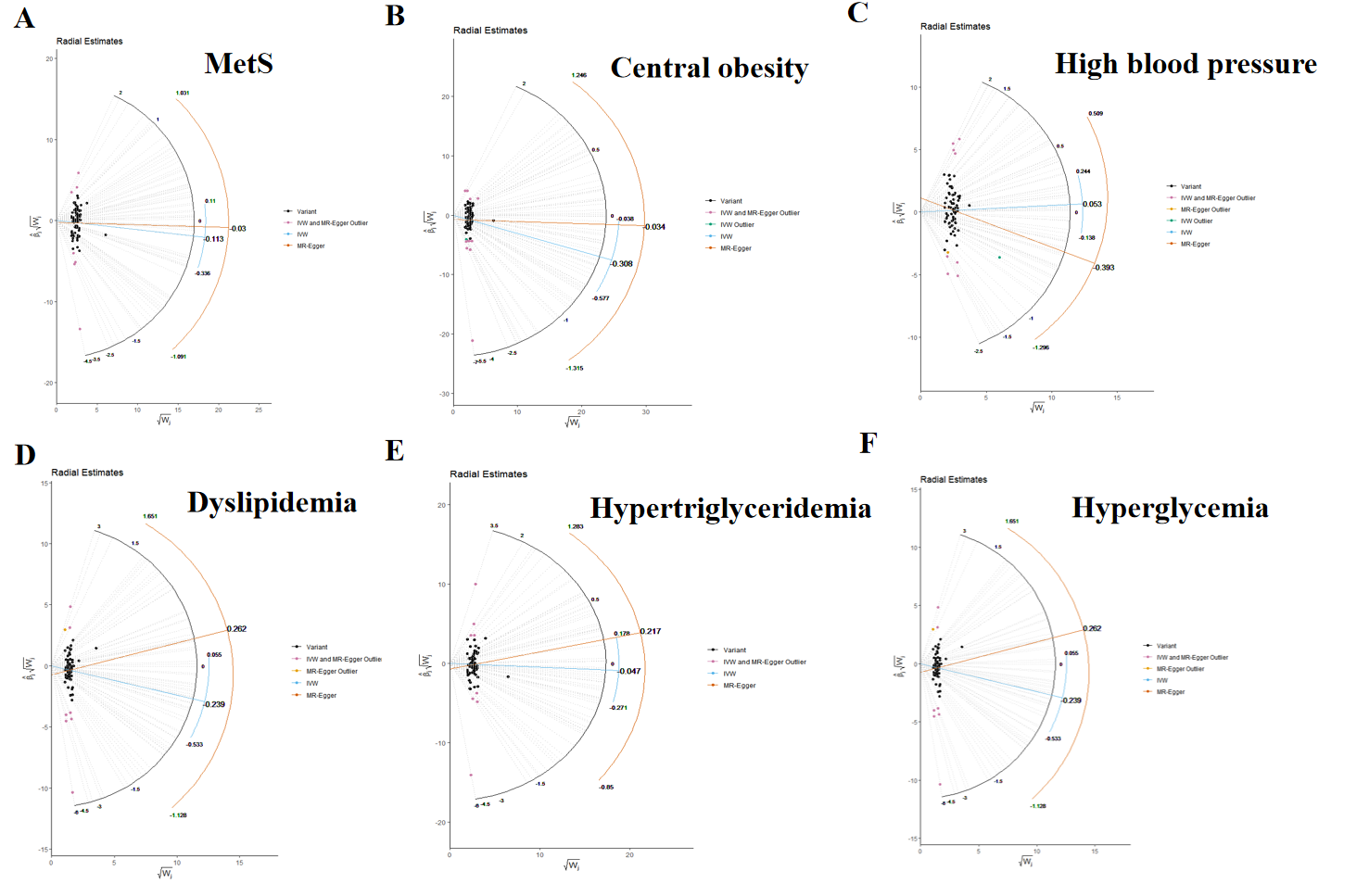


Radial curve means the ratio estimate for each SNP, the overall IVW (in blue) and MR Egger estimate (in orange) identified 7 outlying genetic variants (dots in pink) for MetS, 12 for central obesity, 9 for high blood pressure, 7 for dyslipidemia, 8 for hypertriglyceridemia, and 8 for hyperglycemia.

Outliers are shown in pink with an alpha of 0.05/77 = 6.5E-04.

**Table S10. Associations between genetically predicted one-hour increase in continuous sleep duration and metabolic outcomes using the inverse variance weighted, weighted median, and MR-Egger methods (N = 335,727).**

| **Outcomes** | **Inverse variance weighted** | | **Weighted median** **MR-Egger** | | | |
| --- | --- | --- | --- | --- | --- | --- |
|  | **OR (95% CI)** | ***P* value** | **OR (95% CI)** | ***P* value** | **Intercept*** | ***P* value** |
| **MetS** | **0.89 (0.82, 0.98)** | **0.012** | 1.03 (0.89, 1.21) | 0.674 | -0.003 (-0.016, 0.010) | 0.644 |
| ***Components of MetS*** |  |  |  |  |  |  |
| Central obesity | **0.74 (0.68, 0.80)** | **< 0.0001** | 0.87 (0.74, 1.02) | 0.090 | -0.009 (-0.024, 0.006) | 0.260 |
| High blood pressure | 1.05 (0.96, 1.15) | 0.243 | 1.03 (0.88, 1.21) | 0.682 | 0.002 (-0.009, 0.013) | 0.754 |
| Dyslipidemia | 0.98 (0.89, 1.08) | 0.680 | 1.05 (0.88, 1.25) | 0.574 | -0.002 (-0.017, 0.014) | 0.838 |
| Hypertriglyceridemia | 0.95 (0.88, 1.04) | 0.268 | 0.89 (0.77, 1.02) | 0.092 | -0.005 (-0.018, 0.008) | 0.432 |
| Hyperglycemia | **0.79 (0.68, 0.92)** | **0.002** | 0.89 (0.70, 1.14) | 0.360 | -0.008 (-0.025, 0.008) | 0.340 |

***** MR-Egger intercept was used to quantify the effect of directional pleiotropy. Values that significantly differ from zero indicated potential pleiotropy, which suggested exposure-associated genetic variables may influence the outcome through other pathways rather than through exposure. CI, confidence interval; OR odds ratio; MetS, metabolic syndromes.

**Figure S4. Non-linear** **Mendelian randomization results between genetically predicted continuous sleep duration and metabolic outcomes using piecewise linear method.**


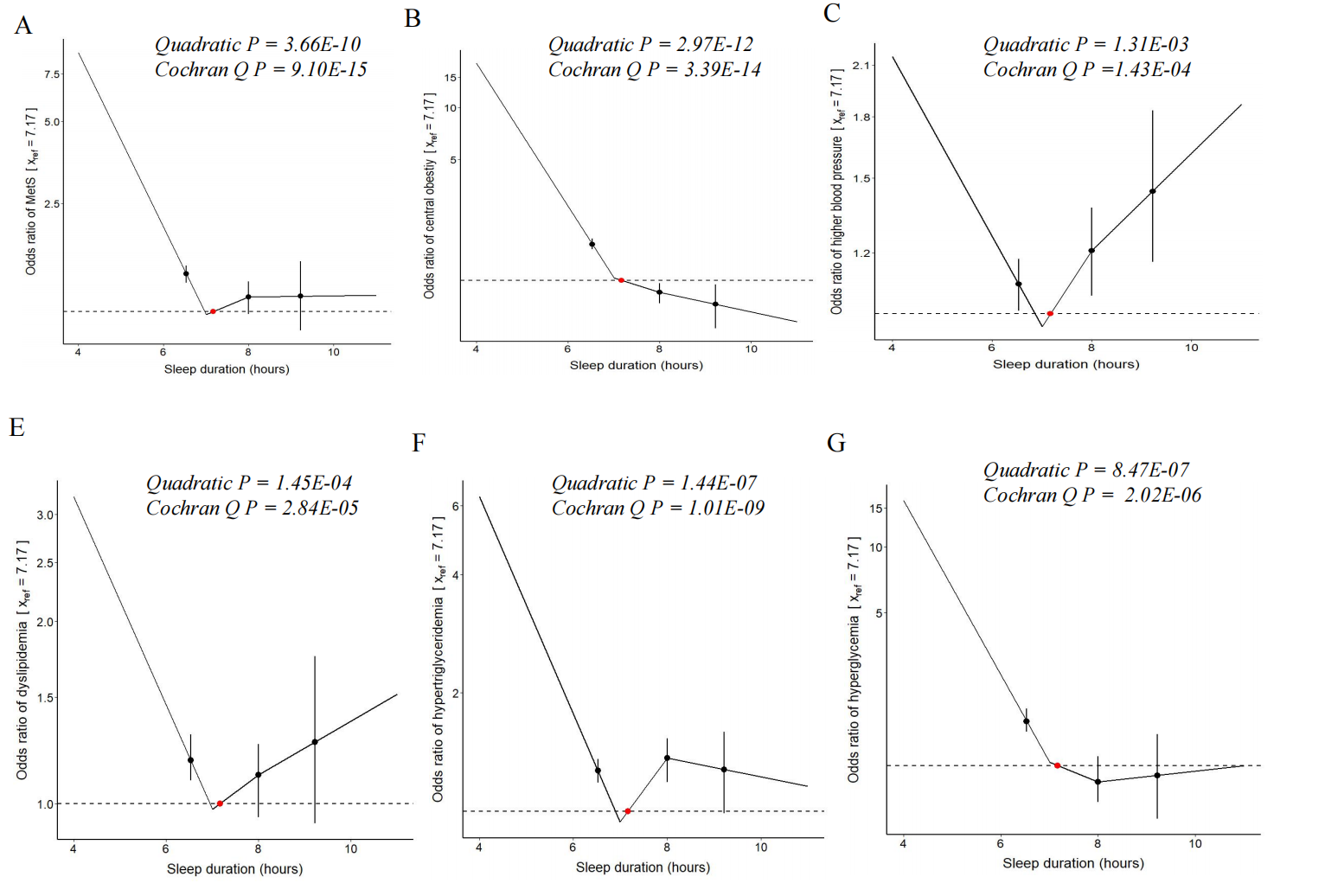


Localized average causal effects (LACE) for MetS and its five components were estimated with piecewise linear method. Black dots (black vertical lines) mean the LACE (95% confidence interval) in each stratum; read dots represent reference point. P quadratic/Cochran Q = P-value for non-linearity from quadratic/Cochran Q test. **(A)** MetS; **(B)** Central obesity; **(C)** High blood pressure; **(D)** Dyslipidemia; **(E)** Hypertriglyceridemia; **(F)** Hyperglycemia.

**Figure S5. Radial Mendelian randomization plots for short sleep duration in metabolic outcomes.**


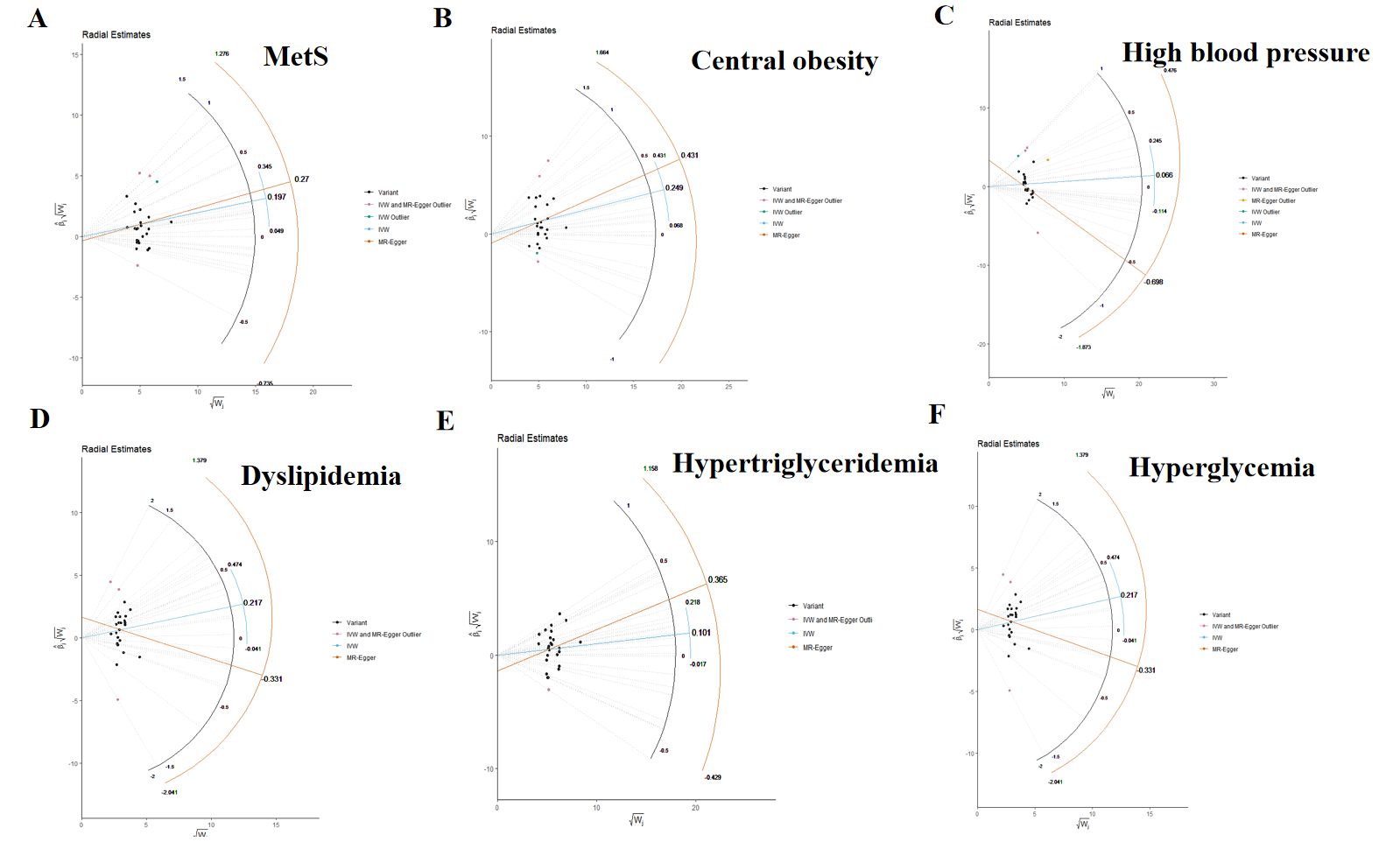


Radial curve means the ratio estimate for each SNP, the overall IVW (in blue) and MR Egger estimate (in orange) identified 3 outlying genetic variants (dots in pink) for MetS, 3 for central obesity, 4 for high blood pressure, 1 for dyslipidemia, 1 for hypertriglyceridemia, and 3 for hyperglycemia. Outliers are shown in pink with an alpha of 0.05/27 = 1.85E-03.

**Table S11. Associations between genetically predicted short sleep duration and metabolic outcomes (N =335,727).**

| **Outcomes** | **Inverse variance weighted** | | **Weighted median MR-Egger*** | | | |
| --- | --- | --- | --- | --- | --- | --- |
|  | **OR (95% CI)** | ***P* value** | **OR (95% CI)** | ***P* value** | **Intercept*** | ***P* value** |
| **MetS** | **1.22(1.13, 1.31)** | **<0.0001** | **1.14 (1.01,1.29)** | **0.030** | 0.001 (-0.020, 0.023) | 0.909 |
| ***Components of MetS*** |  |  |  |  |  |  |
| Central obesity | **1.28 (1.20, 1.38)** | **<0.0001** | **1.11 (0.99, 1.25)** | **0.090** | 0.011 (-0.016, 0.037) | 0.429 |
| High blood pressure | 1.07 (0.99, 1.15) | 0.077 | 0.92 (0.81, 1.05) | 0.209 | 0.020 (-0.005, 0.045) | 0.134 |
| Dyslipidemia | **1.22 (1.12, 1.32)** | **<0.0001** | 0.99 (0.87, 1.12) | 0.830 | -0.0315 (-0.062, -7.3E-04) | 0.057 |
| Hypertriglyceridemia | **1.11 (1.03, 1.18)** | **0.004** | **1.13 (1.01, 1.26)** | **0.037** | -0.004 (-0.013, 0.021) | 0.673 |
| Hyperglycemia | **1.24 (1.09, 1.41)** | **0.001** | **1.38 (1.13, 1.68)** | **0.001** | -0.001 (-0.039, 0.037) | 0.950 |

***** MR-Egger intercept was used to quantify the effect of directional pleiotropy. Values that significantly differ from zero indicated potential pleiotropy, which suggested exposure-associated genetic variables may influence the outcome through other pathways rather than through exposure. CI, confidence interval; OR odds ratio; MetS, metabolic syndromes.

**Figure S6. Radial Mendelian randomization plots for long sleep duration in metabolic outcomes.**


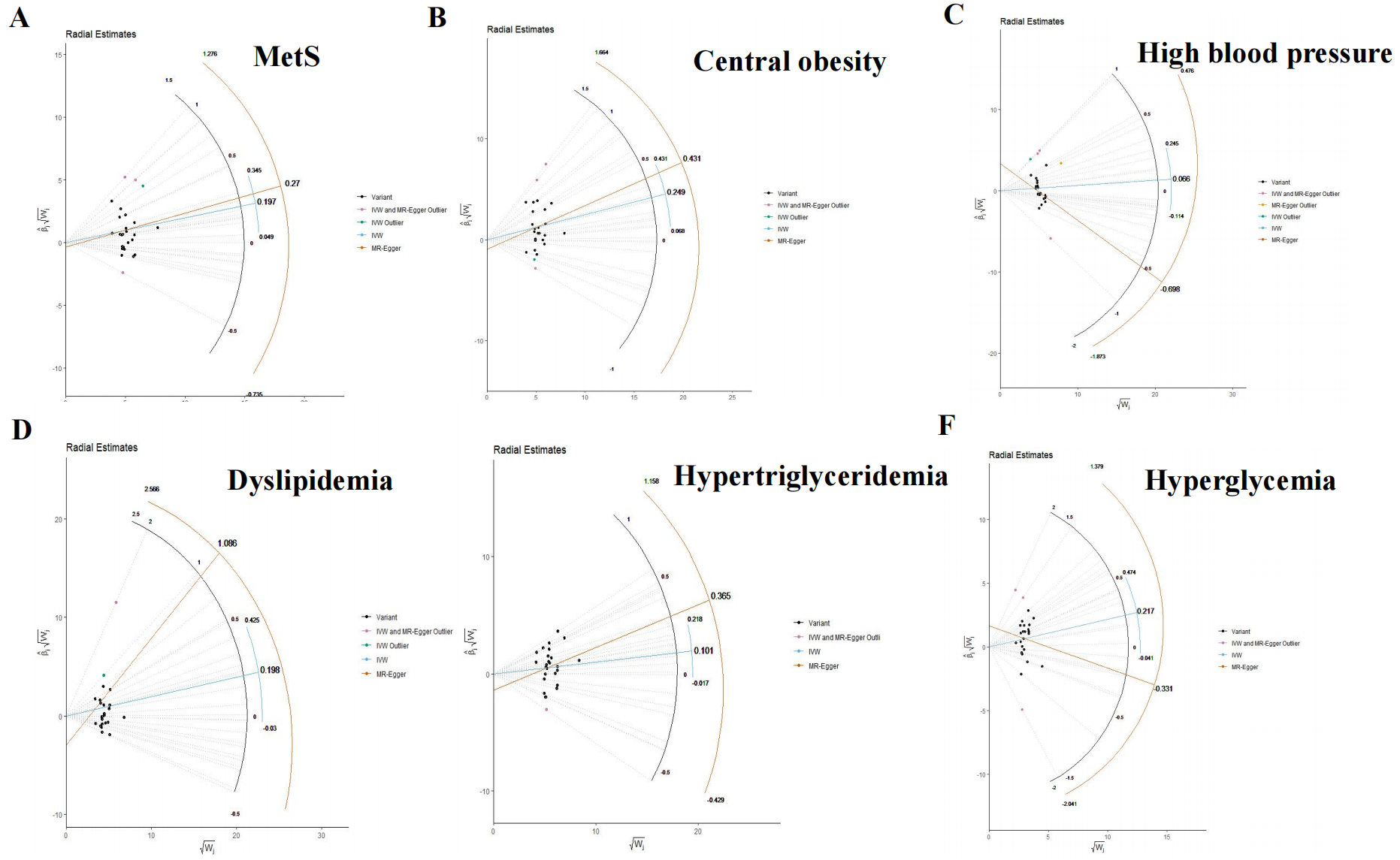


Radial curve means the ratio estimate for each SNP of short sleep duration, the overall IVW (in blue) and MR Egger estimate (in orange) identified 1 outlying genetic variant (dots in pink) for MetS, 2 for central obesity, 2 for high blood pressure,0 for dyslipidemia, 0 for hypertriglyceridemia, and 1 for hyperglycemia. Outliers are shown in pink with an alpha of 0.05/8 = 6.25E-03.

**Table S12. Associations between genetically predicted long sleep duration and metabolic outcomes (N =335,727).**

| **Outcomes** | **Inverse variance weighted** | | **Weighted median MR-Egger** | | | |
| --- | --- | --- | --- | --- | --- | --- |
|  | **OR (95% CI)** | ***P* value** | **OR (95% CI)** | ***P* value** | **Intercept*** | ***P* value** |
| **MetS** | 0.90 (0.82, 0.99) | 0.023 | 1.12 (0.98, 1.28) | 0.105 | -0.046 (-0.105, 0.013) | 0.184 |
| ***Components of MetS*** |  |  |  |  |  |  |
| Central obesity | **0.79 (0.72, 0.87)** | **< 0.0001** | 0.93 (0.82, 1.05) | 0.257 | -0.062 (-0.148, 0.023) | 0.214 |
| High blood pressure | 1.02 (0.93, 1.12) | 0.672 | 1.01 (0.87, 1.18) | 0.885 | 0.002 (-0.052, 0.057) | 0.933 |
| Dyslipidemia | 0.96 (0.87, 1.07) | 0.446 | 1.08 (0.87, 1.19) | 0.814 | -0.035 (-0.065, -0.006) | 0.067 |
| Hypertriglyceridemia | 0.94 (0.86, 1.02) | 0.137 | 1.04 (0.91, 1.18) | 0.572 | -0.022 (-0.049, 0.004) | 0.157 |
| Hyperglycemia | **0.80 (0.69, 0.94)** | **0.008** | 1.08 (0.85, 1.36) | 0.537 | -0.061 (-0.135, 0.012) | 0.164 |

* MR-Egger intercept was used to quantify the effect of directional pleiotropy. Values that significantly differ from zero indicated potential pleiotropy, which suggested exposure-associated genetic variables may influence the outcome through other pathways rather than through exposure. CI, confidence interval; OR odds ratio; MetS, metabolic syndromes.
